# Chemo-Enzymatic Baeyer–Villiger Oxidation Facilitated with Lipases Immobilized in the Supported Ionic Liquid Phase

**DOI:** 10.3390/ma14133443

**Published:** 2021-06-22

**Authors:** Anna Szelwicka, Anna Wolny, Miroslawa Grymel, Sebastian Jurczyk, Slawomir Boncel, Anna Chrobok

**Affiliations:** 1 Department of Chemical Organic Technology and Petrochemistry, Faculty of Chemistry, Silesian University of Technology, Krzywoustego 4, 44-100 Gliwice, Poland; Anna.Szelwicka@polsl.pl (A.S.); Anna.Wolny@polsl.pl (A.W.); 2 Department of Organic Chemistry, Bioorganic Chemistry and Biotechnology, Faculty of Chemistry, Silesian University of Technology, Krzywoustego 4, 44-100 Gliwice, Poland; Miroslawa.Grymel@polsl.pl (M.G.); Slawomir.Boncel@polsl.pl (S.B.); 3 Institute for Engineering of Polymer Materials and Dyes, Lukasiewicz Research Network, Sklodowskiej-Curie 55, 87-100 Torun, Poland; Sebastian.Jurczyk@impib.lukasiewicz.gov.pl

**Keywords:** supported ionic liquid phase, biocatalysis, lipase, chemo-enzymatic Baeyer–Villiger oxidation, heterogeneous catalysis, carbon nanotubes, immobilization

## Abstract

A novel method for chemo-enzymatic Baeyer–Villiger oxidation of cyclic ketones in the presence of supported ionic liquid-like phase biocatalyst was designed. In this work, multi-walled carbon nanotubes were applied as a support for ionic liquids which were anchored to nanotubes covalently by amide or imine bonds. Next, lipases B from *Candida antarctica*, *Candida rugosa,* or *Aspergillus oryzae* were immobilized on the prepared materials. The biocatalysts were characterized using various techniques, like thermogravimetry, IR spectroscopy, XPS, elemental analysis, and SEM-EDS microscopy. In the proposed approach, a biocatalyst consisting of a lipase as an active phase allowed the generation of peracid in situ from the corresponding precursor and a green oxidant–hydrogen peroxide. The activity and stability of the obtained biocatalysts in the model oxidation of 2-adamantanone were demonstrated. High conversion of substrate (92%) was achieved under favorable conditions (toluene: *n*-octanoic acid ratio 1:1 = v:v, 35% aq. H_2_O_2_ 2 eq., 0.080 g of biocatalyst per 1 mmol of ketone at 20 °C, reaction time 4 h) with four reaction cycles without a drop in its activity. Our ‘properties-by-design’ approach is distinguished by its short reaction time at low temperature and higher thermal stability in comparison with other biocatalysts presented in the literature reports.

## 1. Introduction

Due to the growing significance of sustainable processes in the chemical industry, there is a constantly increasing interest in biocatalysis [1]. Biocatalysis is a useful tool enabling the production of fine chemicals, i.e., pharmaceuticals, polymers, textiles, biofuels, food, surfactants, or perfumes [2,3]. Mild reaction conditions, high selectivity, and hence low-waste processes constitute the key results of the enzyme performance. The weak points are low thermal- and chemical stability of the biocatalyst under typical technological conditions. Organic compounds and solvents as well as higher temperatures negatively affect the enzymes’ activity [4].

Various strategies were used to prevent enzyme deactivation but one of the most used methods for enzyme stabilization is the immobilization on the surface of solid carrier via covalent and non-covalent bonds [5,6,7]. Various materials can be used here as carriers, i.e., carbon nanotubes (CNTs), silica materials, metal oxides, polymers, clay materials, or hybrid materials connecting features of the component materials [8]. Due to the exceptional properties, i.e., designable surface area, high volume/size ratio, significant mechanical and thermal stability, insolubility in a reaction environment, durability upon acidic or basic conditions, and simplicity of functionalization, CNTs were found to be excellent enzyme supports [9,10]. As mentioned, to improve stability of the enzyme, CNTs can be modified by non-covalent interactions (physical adsorption, electrostatic forces, π-π interactions, van der Waals forces, hydrophobic interactions) or covalent bonds (via or without linkers) [11]. Formation of the heterogeneous materials facilitates handling, separation and durability of the biocatalyst. Furthermore, immobilization of lipases on a hydrophobic surface increases their activity due to the phenomenon called ‘interfacial activation’ [12,13,14].

The high performance of lipase B from *Candida antarctica* (CALB) immobilized on the surface of CNTs was confirmed in several applications in our previous studies. The modification of solid carrier equipped with organic groups or other modifiers, e.g., polytetrafluoroethylene or ionic liquids is a commonly used method to match the properties of the surface to the enzyme requirements [15,16,17,18,19,20].

Ionic liquids (ILs) are fully ionized species of the unique properties such as negligible vapor pressure and the potential to maintain enzymes in their active conformation [21,22]. ILs with their organized ‘nanostructures,’ hydrogen-bonded polymeric supramolecules, as well as polar and non-polar regions, have been effectively applied in bioprocesses as reaction media and immobilization agents [23,24,25]. Hence, due to the versatility of their actions and structure, ILs were recently utilized not only as an enzyme’s stabilizing agent [21,22,23,24,25], but also in other applications, i.e., extraction [26], catalysis [27], fuel cells [28], inhibition of tumor growth [29], or gas absorption [30]. ILs can be immobilized on the solid support in the form of a thin layer reducing, at the same time, the amount of IL used in the process (SILP, supported ionic liquid phase) [31]. As a result, a solid heterogeneous material is obtained which facilitates recyclability and stability of the biocatalyst. On the other hand, in the micro-scale, enzymes can be captured in the layer of IL. In addition, ionic species can be anchored to a solid support via covalent bonds, forming supported ionic liquid-like phase (SILLP) [25,31,32]. SILP/SILLP were used for the immobilization of enzymes and the biocatalysts were next applied in hydrolysis [33], transesterification [34], and esterification [31,35] reactions. Only few works described the use of CNTs for SILLP biocatalysis [33,36]. For instance, ILs were covalently immobilized on the surface of MWCNTs serving as the support for CALB and, next, used as biocatalyst in the hydrolysis of triacetin. Some other improvements in the stability and reusability were achieved but still slow leaching of the enzyme from the SILP carrier was observed.

Design of the stable biocatalyst for the chemo-enzymatic oxidation using H_2_O_2_ as an oxidant is a challenge due to the detrimental effect of peroxy species on the enzymes, called in the literature ‘dangerous liaison’. In many examples the exposure of a lipase to high concentration of aq. hydrogen peroxide resulted in its partial or complete deactivation. Hydrogen peroxide can react with proteins and the formation of protein derivatives or peptide fragments possessing highly reactive carbonyl groups (ketones, aldehydes) was observed so far [37,38,39]. Here, the model Baeyer–Villiger oxidation reaction was chosen. The idea of using hydrogen peroxide aimed at the practical probing of the biocatalyst performance under harsh reaction conditions. Baeyer–Villiger oxidation of cyclic ketones is a commonly used reaction for the synthesis of lactones which are utilized, i.e., as monomers in the polymer industry, in the synthesis of pharmaceuticals, fragrances or solvents [38,39]. In the chemo-enzymatic approach, the enzymes are responsible for the in situ generation of the oxidizing agent which is subsequently utilized in the main step of the process. The method is based on the generation of peracid in situ from a peracid precursor (short–medium-chain carboxylic acids or ethyl acetate) in the presence of lipases, using hydrogen peroxide as the primary oxidant. The most used enzyme in this process is CALB—in either native or immobilized forms: Novozyme 435, cross-linked enzyme aggregates, immobilized on mesoporous modified silica [40,41,42,43,44,45,46,47] or on CNTs [16].

In this study, the chemo-enzymatic Baeyer–Villiger oxidation of cyclic ketones to lactones—in the presence of SILLP biocatalysts containing CALB, lipase from *Candida rugosa* (CRL) or lipase from *Aspergillus oryzae* (AOL)—was presented for the first time. To our best knowledge, this work presents the first successful attempt of implementation of SILP biocatalyst under extremely harsh conditions for lipases, that is in the presence of hydrogen peroxide in the chemo-enzymatic pathway of Baeyer–Villiger oxidation. We have cross-verified and proved their exceptional catalytic properties hence the elaborated approach fully corresponds to the sustainable development of the process.

## 2. Materials and Methods

### 2.1. Materials

2-Adamantanone (purity min. 99%), solvents (purity min. 99.5%), dioctyl sulfate (purity 99%), trioctyl phosphate (purity 98%), 1-butyl-3-methylimidazolium dioctyl phosphate (purity 98%), lithium bis(trifluoromethylsulfonyl)amide (purity 99%), sodium dicyanamide (purity 99%), *N*-(3-aminopropyl)imidazole (purity min. 98%), 1-iodohexane (purity 98%), thionyl chloride (purity 99%), dichloromethyl methyl ether (purity min. 98%), *n*-octanoic acid (purity 99%), and *n*-decane were purchased from Chemat, Gdansk, Poland. Titanium tetrachloride (purity 99%), native lipase B from *Candida antarctica* in aqueous-glycerol solution (activity 5000 U∙L∙kg^−1^), lyophilized lipase from *Candida rugosa* (activity 700 U∙L∙kg^−1^) and native lipase from *Aspergillus oryzae* in aqueous-glycerol (activity 20,000 U∙L∙kg^−1^) were purchased from Sigma-Aldrich (Merck Group, Warsaw, Poland). 1-Butyl-3-methylimidazolium bis(trifluoromethylsulfonyl)amide (purity 98%) and 1-butyl-3-methylimidazolium dibutyl phosphate (purity 98%) were purchased from IoLiTech Ionic Liquids Technologies GmbH, Heilbronn, Germany. Industrial grade pristine (CNTs) and oxidized (CNTs-COOH) MWCNTs were purchased from CheapTubes Inc. (Grafton, VT, USA).

### 2.2. Methods

GC-FID analyses were performed using a Shimadzu GC-2010 Plus equipped with a Zebron ZB 5MSi column (30 m × 0.32 mm × 0.25 μm thickness of film) (Phenomenex, Warsaw, Poland). GC-MS analysis was performed using Agilent Technologies 7890C equipped with a mass spectrometer Agilent Technologies 5975C detector (Agilent Technologies Schweiz AG, Basel, Switzerland) with HP 5MS column (30 m × 0.25 mm × 0.25 μm) (Phenomenex, Warsaw, Poland) and electron impact (EI) ionization at 70 eV (ESI—Section S1).

^1^H NMR and ^13^C NMR spectra were recorded on a Varian system (600 MHz and 151 MHz, respectively) (ESI—Section S2) (Palo Alto, California, USA).

Scanning electron microscopy (SEM) images were obtained using a Phenom Pro Desktop SEM instrument with an EDS detector (15 kV) (Thermo Fischer Scientific, Warsaw, Poland) (ESI—Section S3).

TG analysis was performed applying Mettler Toledo TGA851e thermobalance (Columbus, Ohio, USA). The samples (approximately 10 mg) were heated in a range of 25–800 °C and the rate was set at 10 °C min^−1^. The thermograms were performed in a reference to standard (70 μL Al_2_O_3_ crucibles) under a dynamic nitrogen flow set at 60 mL/min (ESI—Section S4).

Infrared spectra were recorded on a Nicolet 6700 FT-IR spectrophotometer (Thermo Fischer Scientific, Warsaw, Poland).The operating conditions were as follows: KBr pellet—0.1 mg CNTs/1 g KBr, wavelength range of 400–4000 cm^−1^, 16 scans, resolution of 4 cm^−1^ (ESI—Section S5).

XPS measurements were performed using a VG Scientific photoelectron spectrometer ESCALAB-210 (Thermo Fischer Scientific, Warsaw, Poland) employing Al Kα (1486.6 eV) X-ray source operated at 300W. During measurements, the pressure was set at approximately 5∙10^−9^ mbar. Survey spectra was recorded for the sample in the energy range from 0 to 1350 eV with 0.4 eV step and 75 eV analyzer pass energy. High resolution spectra were recorded with 0.1 eV step, 100 ms dwell time, and 20 eV pass energy. A ninety degree take-off angle was used in all measurements. The curve fitting was performed using the Avantage (ver. 4.84) software provided by Thermo Electron (Thermo Fischer Scientific, Warsaw, Poland), which describes each component of the complex envelope as a Gaussian–Lorentzian sum function; a constant 0.3 (±0.05) G/L ratio was used. The background was fitted using nonlinear Shirley model. Scofield sensitivity factors and measured transmission function were used for quantification (ESI—Section S6).

Elemental analysis (EA) was performed on a Vario MACRO (Elementar Analysesysteme GmbH, Germany) using VARIOEL 5.14.4.22 software (ESI—Section S7).

The presence of lipase in the filtrate after 5 reaction cycles was determined via Lowry’s protein detection method (using UV-Vis spectroscopy) according to the literature [18]. UV-Vis spectra were recorded on a Jasco V-650 spectrophotometer (Jasco, Cracow, Poland) at 25 °C in an aqueous solution and the absorbance values (wavelength λ = 670 nm) was recorded.

### 2.3. Synthetic Procedures

#### 2.3.1. Synthesis of CNTs-CONH (A1)

Into a 250 mL three-neck round-bottom flask, 10 g of CNTs-COOH was introduced. Next, the content was tightly sealed and purged by an inert gas (argon). Afterwards, 150 mL of thionyl chloride was added dropwise and the reaction was carried out for 24 h under reflux in an inert atmosphere. After this time, CNTs-COCl-containing mixture was cooled down, CNTs-COCl were filtered off, washed with anhydrous tetrahydrofuran (10 × 50 mL), and dried under vacuum on the Schlenk line (24 h, 25 °C, 1 mbar). Prepared CNTs-COCl material was introduced into the step of synthesis of the amide bond according to the literature [33]. CNTs-COCl (10 g) was introduced to the 500 mL three-neck round-bottom flask and tightly sealed. Next, 200 mL of *N*-(3-aminopropyl)imidazole was added. The mixture was stirred at 120 °C for 24 h, then the solid was cooled down, filtered off, washed with anhydrous acetonitrile (8 × 50 mL) and dichloromethane (8 × 50 mL). Final CNTs-CONH **(A1)** was next dried on the Schlenk line (24 h, 25 °C, 1 mbar). The obtained material was analyzed using thermogravimetry, elemental analysis, FT-IR spectroscopy, XPS and SEM-EDS microscopy (ESI, Figures S4 and S45; Table S2; Sections S5 and S7).

#### 2.3.2. Synthesis of CNTs-C=N (I1)

The synthesis was carried out according to the literature [48]. Into a 500 mL three-neck round-bottom flask, 10 g of pristine CNTs, 15 mL of dichloromethyl methyl ether and 200 mL of anhydrous dichloromethane were introduced. The mixture was cooled down to 0 °C. The flask content was tightly sealed and purged by an inert gas (argon), then 15 mL of TiCl_4_ was added dropwise and the mixture was stirred for 1 h. Next, the mixture was heated up to 25 °C and stirred for 24 h. Then, the solid was filtered off, washed with deionized water (200 mL) and acetone (250 mL) until achieving a colorless liquid. The CNTs-CHO material was next dried on the Schlenk line (24 h, 60 °C, 1 mbar). Then, 10 g of CNTs-CHO material was suspended in dichloromethane (100 mL) and introduced to the 500 mL round-bottom flask according to the literature [49]. The mixture was cooled down to 0 °C and then 100 mL of *N*-(3-aminopropyl)imidazole was added. The content was stirred for 24 h at 25 °C. Then, the solid was filtered off, washed with 200 mL of dichloromethane and dried on the Schlenk line (24 h, 25 °C, 1 mbar). The final CNTs-C=N material **(I1)** was characterized using thermogravimetry, elemental analysis and FT-IR spectroscopy (ESI, Sections S5 and S7).

#### 2.3.3. Synthesis of CNTs-CONH-HEX-I (A2) and CNTs-C=N-HEX-I (I2)

Into a 50 mL round-bottom flask, 2 g of CNTs-**CONH (A1) or CNTs-C=N (I1)** and 20 mL of 1-iodohexane were introduced. The mixtures were stirred for 24 h at 110 °C and then cooled down, filtered off, and washed with acetonitrile (8 × 25 mL) and dichloromethane (4 × 25 mL). The final structures after quaternization: CNTs-CONH-HEX-I **(A2)** or CNTs-C=N-HEX-I **(I2)** were next dried on the Schlenk line (24 h, 25 °C, 1 mbar) and analyzed using thermogravimetry, elemental analysis, XPS and SEM-EDS microscopy (ESI, Figures S5, S10, S46 and S48; Tables S3 and S5; Section S7).

#### 2.3.4. Synthesis of CNTs-CONH-HEX-N(Tf)_2_ (A3) and CNTs-C=N-HEX- N(Tf)_2_ (I3)

Into a 50 mL round-bottom flask, 1 g of CNTs-CONH-HEX-I (A2) or CNTs-C=N-HEX-I (I2) in 15 mL of dichloromethane were introduced. Then, 3 g of lithium bis(trifluoromethylsulfonyl)amide dissolved in 15 mL of deionized water was added dropwise. The mixtures were stirred for 24 h at 25 °C, the resulted solids were filtered off, washed with deionized water (6 × 25 mL—until the absence of iodide—test with AgNO_3_), dichloromethane (4 × 25 mL), and dried on the Schlenk line (24 h, 25 °C, 1 mbar). The obtained CNTs-CONH-HEX-N(Tf)_2_ **(A3)** or CNTs-C=N-HEX-N(Tf)_2_ **(I3)** materials were analyzed via thermogravimetry, elemental analysis and SEM-EDS microscopy (ESI, Figures S6, S11, Section S7).

#### 2.3.5. Synthesis of CNTs-CONH-HEX-N(CN)_2_ (A4) and CNTs-C=N-HEX- N(CN)_2_ (I4)

Into a 50 mL round-bottom flask, 1 g of CNTs-CONH-HEX-I (A2) or CNTs-C=N-HEX-I (I2) in 15 mL of dichloromethane were introduced. Then, 2.5 g of NaN(CN)_2_ dissolved in 15 mL of deionized water was added dropwise. The mixtures were stirred for 24 h at 25 °C, the resulted solids were filtered off, washed with deionized water (4 × 25 mL), dichloromethane (4 × 25 mL), and dried on the Schlenk line (24 h, 25 °C, 1 mbar). The obtained CNTs-CONH-HEX-N(CN)_2_ **(A4)** or CNTs-C=N-HEX-N(CN)_2_ **(I4)** materials were analyzed via thermogravimetry, elemental analysis and SEM-EDS microscopy (ESI, Figures S7 and S12, Section S7).

#### 2.3.6. Synthesis of CNTs-CONH-Oc_2_PO_4_ (A5) or CNTs-C=N- Oc_2_PO_4_ (I5)

Into a 50 mL round-bottom flask, 2 g of CNTs-CONH (A1) or CNTs-C=N (I1) and 25 mL of trioctyl phosphate were introduced. The mixtures were stirred for 24 h at 25 °C, the resulted solids were filtered off, washed with acetonitrile (4 × 25 mL) and dichloromethane (4 × 25 mL), and dried on the Schlenk line (24 h, 25 °C, 1 mbar). The obtained CNTs-CONH- Oc_2_PO_4_ **(A5)** or CNTs-C=N- Oc_2_PO_4_ **(I5)** materials were analyzed via thermogravimetry, elemental analysis, and SEM-EDS microscopy (ESI, Figures S8, S13, and S18; Section S7).

#### 2.3.7. Synthesis of CNTs-CONH-OcSO_4_ (A6) or CNTs-C=N- OcSO_4_ (I6)

Into a 50 mL round-bottom flask, 2 g of CNTs-CONH (A1) or CNTs-C=N (I1) and 20 mL of dioctyl sulfate were introduced. The mixtures were stirred for 24 h at 25 °C, the resulted solids were filtered off, washed with acetonitrile (4 × 25 mL) and dichloromethane (4 × 25 mL) and dried on the Schlenk line (24 h, 25 °C, 1 mbar). The obtained CNTs-CONH- OcSO_4_ **(A6)** or CNTs-C=N- OcSO_4_ **(I6)** materials were analyzed via thermogravimetry, elemental analysis and SEM-EDS microscopy (ESI, Figures S9 and S14, Section S7).

#### 2.3.8. Synthesis of CNTs-CONH-[bmim][N(Tf)_2_] and CNTs-C=N-[bmim][N(Tf)_2_]

Into a 50 mL round-bottom flask, CNTs-CONH (A1) or CNTs-C=N (I1) (1.00 g), acetonitrile (25 mL) and [bmim][N(Tf)_2_] (0.1–1.1 g) were introduced. The suspension was stirred for 24 h at 25 °C on a magnetic stirrer (800 rpm), and obtained solids were filtered off, washed with acetonitrile (4 × 25 mL), and dried on the Schlenk line (1 mbar, 25 °C, 24 h).

#### 2.3.9. Immobilization of the Lipase

The immobilization step was carried out according to the literature [15,16,17,18,19,20]. Aqueous-glycerol solutions of native lipases: lipase B from *Candida antarctica*, lipase from *Aspergillus oryzae* and lyophilized lipase from *Candida rugosa* (7.5 g), a CNTs-CONH- or CNTs-C=N-based support (1.0 g) and deionized water (30 mL) (pH in a range of 6.20–6.30) were introduced into a 100 mL round bottom flask and then stirred in a thermostatic shaker (250 rpm) at 20 °C for 3 h. Next, the biocatalyst was filtered off under vacuum and washed with water (4 × 50 mL). The obtained biocatalysts were dried over anhydrous P_2_O_5_ in a desiccator at 4 °C for 3 days.

#### 2.3.10. General Procedure of the Baeyer–Villiger Oxidation of 2-Adamantanone

Into a 25 mL round-bottom flask, 2-adamantanone (1 mmol), a biocatalyst (0.02 g–0.10 g), n-octanoic acid (0.5 mL), toluene (0.5 mL), and n-decane (20 wt.% in relation to the ketone; internal standard for GC analysis) were successively introduced. Next, aq. 35 wt.%. solution of hydrogen peroxide (2 eq. in relation to the ketone, 2 mmol, 0.194 g) was added dropwise. The flask was sealed with a septum and stirred in a thermostatic shaker (400 rpm) at 20−60 °C for 1−24 h, depending on the reaction rate. During the reaction, 40 μL of the samples diluted with 1.5 mL of acetonitrile were collected, in order to track the reaction progress using GC apparatus. 

Characterization of the product (4-oxatricyclo[4.3.1.13.8]undecan-5-one): ^1^H NMR (600 MHz, CDCl_3_): δ_H_ 1.70–2.15 (m, 12H), 3.01–3.12 (m, 1H), 4.39–4.52 (m, 1H) ppm. ^13^C NMR (151 MHz, CDCl_3_): δ_C_ 25.8, 30.9, 33.7, 35.7, 41.2, 73.1, 178.9 ppm. EI (m/z (%)): 166 (2), 122 (5), 107 (18), 93 (18), 80 (100), 67 (16), 53 (28), 41 (17).

#### 2.3.11. Recycle of the Biocatalyst

After the reaction run, the biocatalyst was filtered off, washed with 20 mL of hexane, and dried under vacuum on the Schlenk line (8 h, 25 °C, 1 mbar). Next, the biocatalyst was introduced to the following reaction run.

## 3. Results and Discussion

### 3.1. Synthesis and Characterization of Biocatalysts

Supported ionic liquid-like phase technique concerns the modification of a structure of a solid support towards obtaining ionic compounds covalently bonded to its surface. Hence, in these studies multi-walled carbon nanotubes oxidized to carboxylic or formyl groups were next modified by aminoalkyloimidazole-derivative towards synthesis of an aromatic cation precursor, and then various hydrophobic and hydrophilic anions were introduced to the final structures. Cations were anchored to the support via amide or imine bond, and as anions the following structures were selected: hydrophilic: [Oc_2_PO_4_]^−^ and [OcSO_4_]^−^, and hydrophobic bis(trifluoromethylsulfonyl)amide [N(Tf)_2_]^-^ and dicyanamide [N(CN)_2_]^−^. Diversity of the structure of anions allowed us to better understand their influence on the protein’s activity and stability in the enzymatic and chemo-enzymatic reactions.

**Scheme 1:**
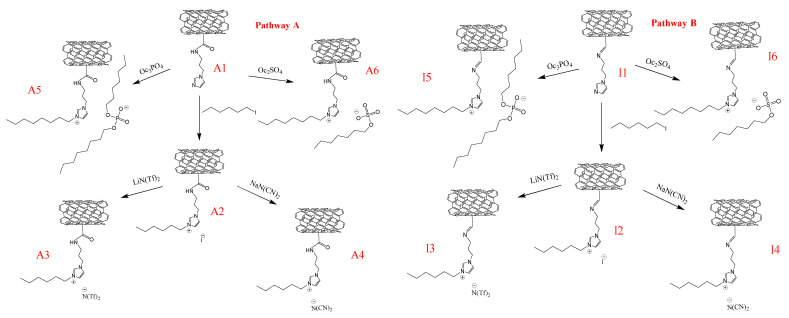
The synthesis of CNTs-CONH-cored (pathway A) and CNTs-C=N-cored (pathway B) SILLP carriers.

The first step of the synthesis of biocatalyst was assumed on the covalent bonding of *N*-(3-aminopropyl)imidazole—as the cation precursor—on the surface of MWCNTs. To this aim, the surface of MWCNTs was modified with carboxylic (CNTs-COOH—commercially available, content of functional groups: 1.4 wt.%) of formyl groups (CNTs-CHO, content of functional groups: 0.7 wt.%) using the procedure described in the literature [48]. As a result, the amide bond formed by the reaction between -COOH and -NH_2_ groups from the alkyl chain of the substituted imidazole (CNTs-CONH (**A1)**, functional groups 5.8 wt.%), and, respectively, the imine bond between -CHO and -NH_2_ groups (CNTs-C=N **(I1)**, functional groups 3.2 wt.%) (Scheme 1).

The imidazolium ring was subsequently quaternized using 1-hexyl iodide (CNTs-CONH-HEX-I **(A2)**, CNTs-C=N-HEX-I (**I2)**), or modified via alkylation using octyl esters of sulfuric or phosphoric acids (CNTs-CONH- Oc_2_PO_4_ **(A5)**, CNT-CONH- OcSO_4_ **(A6);** CNTs-C=N- Oc_2_PO_4_ **(I5)**, CNTs-C=N- OcSO_4_ **(I6)**). An exothermic alkylation using esters of sulfuric or phosphoric acid were carried out at room temperature for 24 h in a solventless system. Octyl chain was used to design a hydrophobic region in a structure, privileged for the lipase activation. On the other hand, 1-hexyl iodide was used to introduce a hydrophobic medium-length linear alkyl chain to the cation. This synthesis was carried out for 24 h under reflux, using the quaternized agent as the solvent. The supports with iodide anion were then subjected to anion-exchange reactions with Li[N(Tf)_2_] or Na[N(CN)_2_] carried out in a biphasic water/DCM system at room temperature, resulting in the final SILLP carriers formation (CNTs-CONH-HEX-N(Tf)_2_ **(A3),** CNTs-CONH-HEX-N(CN)_2_ **(A4)**; CNTs-C=N-HEX-N(Tf)_2_ **(I3)**, and CNTs-C=N-HEX-N(CN)_2_ **(I4)**).

The synthesized materials were fully characterized using various techniques: thermogravimetric analysis (TG) (ESI- Section S4), scanning electron microscopy (SEM) equipped with energy-dispersive detector (EDS) (ESI- Section S3), elemental analysis (EA) (ESI- Section S7), X-ray photoelectron spectroscopy (XPS) (ESI- Section S6) and Fourier transform infrared spectroscopy (FT-IR) (ESI- Section S5). Calculated mass content of functional groups (TG) and lipases after immobilization (TG) on all SILLP supports was presented in Table 1.

**Scheme 2:**
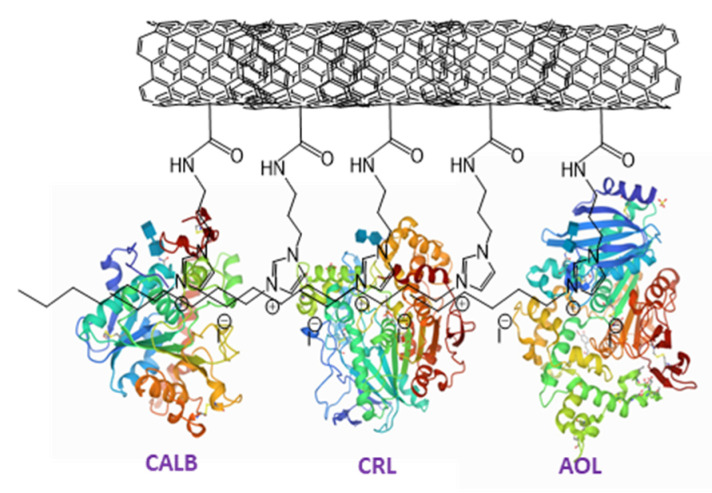
An exemplary structure of the biocatalyst used in this study.

The last step of biocatalysts synthesis was the adsorption of enzymes: CALB, lipase from *Candida rugosa* (CRL) or lipase from *Aspergillus oryzae* (AOL) on the various types of supports (Scheme 2). To this aim, the supports (**A2-A6** and **I2-I6**) and an enzyme were dispersed in deionized water (mass ratio of the enzyme to support 7.5:1) and stirred in a thermostatic shaker for 3 h. The final biocatalyst was filtered off washed with deionized water and dried over anhydrous P_2_O_5_. The amount of an enzyme adsorbed on the surface of SILLP (0.6–9.2 wt.%) was determined by TGA (Table 1).

The results varied and strongly depended on the type of an anion introduced to the support, the linking bond of IL to the surface of MWCNTs as well as the type of a lipase. The highest amount of an adsorbed lipase was obtained once the hydrophobic anion i.e., N(Tf)_2_ or N(CN)_2_ were involved to immobilize CRL or AOL. However, the results observed for CALB revealed that higher amount of an enzyme was adsorbed on the surface of a support if hydrophilic anions were used instead. Nevertheless, these results depended on the type of additives/stabilizers added to the commercially available enzymatic preparations. Their presence can change the affinity of a lipase to the support upon immobilization conditions as well. In addition, the structure of each lipase differs, which has a significant influence on the immobilized amount. Linking the ILs cation via imine bond leads to the increase in affinity of CRL and AOL to the support, while in the case of CALB oppositely. This outcome might be driven by the lower degree of functionalization of the starting material (Table 1), hence the further hydrophobicity of final material slightly varied; even if IL with a similar anion was applied. This phenomenon can be explained by a molecular insight. Due to the structure of an active center of CALB, which is incompletely covers by externally hydrophilic/internally hydrophobic lid, their properties and affinity depend much less on the external factors in comparison with two other lipases with fully covered active centers, which leads to its weaker differentiation in the affinity to the carrier [12,50,51,52,53,54].

**Scheme 3:**
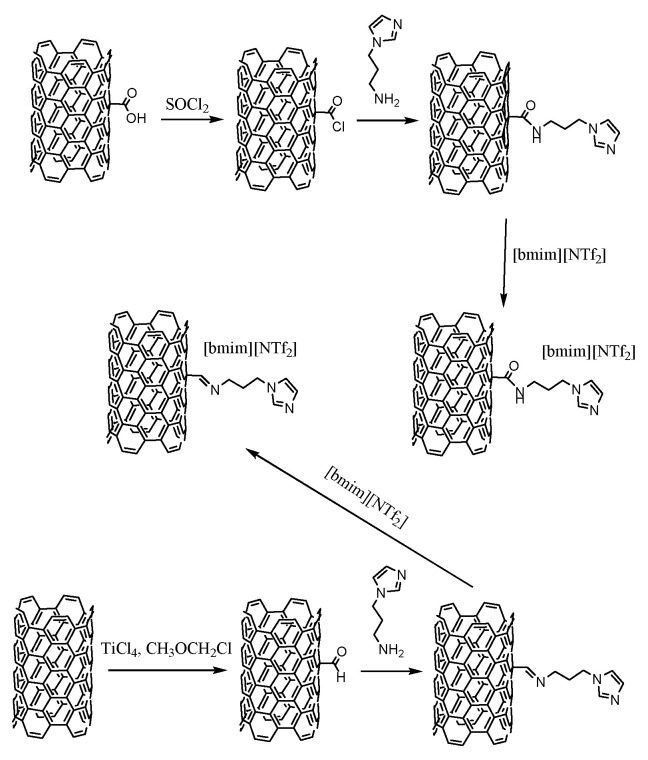
Preparation of SILP-[bmim][NTf_2_]-based supports.

For comparison, the physical adsorption of [bmim][N(CN)_2_] ionic liquid (1-butyl-3-methylimidazolium cation) on non-ionic materials: CNTs-CONH **(A1)** and on CNTs-C=N **(I1)** was performed (Scheme 3). This approach was tested due to enzyme’s stabilizing properties of ILs, hence it was predicted that immobilization of an ionic liquid on the CNTs-CONH and CNTs-C=N supports allow to obtain more active and stable biocatalyst in comparison with previously presented SILLPs-based biocatalysts. The SILP biocatalysts based on both CNTs-CONH **(A1)** and on CNTs-C=N **(I1)** materials (support: IL mass ratio 1: 0.1–1.1) were obtained as the consequence and characterized using thermogravimetry (Table 2). The ionic liquid used to stabilize an enzyme was carefully selected based on our previous studies related to the stabilization of a native CALB in Baeyer–Villiger oxidation [46]. Application of SILP technique allows to reduce the amount of IL in the process with subsequent remaining its stabilizing properties [25,31,32]. The lipase was immobilized in the same way as the protocol described above.

### 3.2. SILLP Biocatalysts in Chemo-Enzymatic Baeyer–Villiger Oxidation

**Scheme 4:**
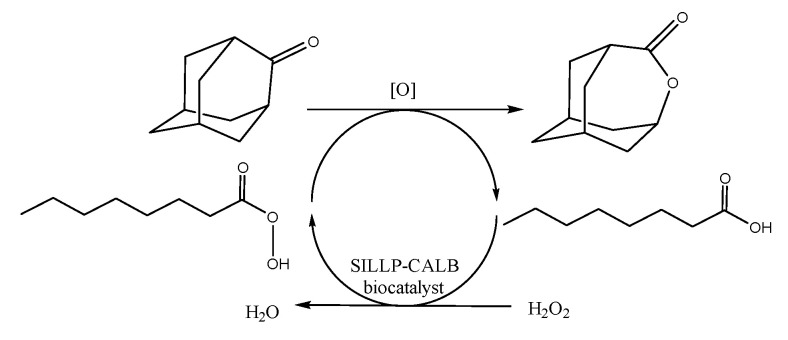
Oxidation of 2-adamantanone to 4-oxatricyclo[4,3,1,13,8]undecan-5-one via chemo-enzymatic protocol.

Catalytic performance of SILLP biocatalysts was tested in the chemo-enzymatic Baeyer–Villiger oxidation of cyclic ketones with 2-adamantanone as the model ketone (Scheme 4). The model reaction was selected based on relatively high reactivity of the ketone and the exceptional selectivity of lactone (over 99%) in chemo-enzymatic protocol.

The initial process conditions were selected based on our previous studies related to the application of CALB immobilized on multi-walled carbon nanotubes in chemo-enzymatic Baeyer–Villiger oxidation [16]. The reaction was carried out at 20 °C with 35% aq. H_2_O_2_ as the oxidant (2:1 molar ratio to ketone), *n*-octanoic acid (3.2:1 molar ratio to the ketone) as a peracid precursor and toluene as a solvent. Of each biocatalyst, 0.080 g was used per 1 mmol of ketone regardless of enzyme content in biocatalyst to performed the comparison studies of the activity.

#### 3.2.1. Influence of the Structure of SILLP Biocatalyst

**Figure 1:**
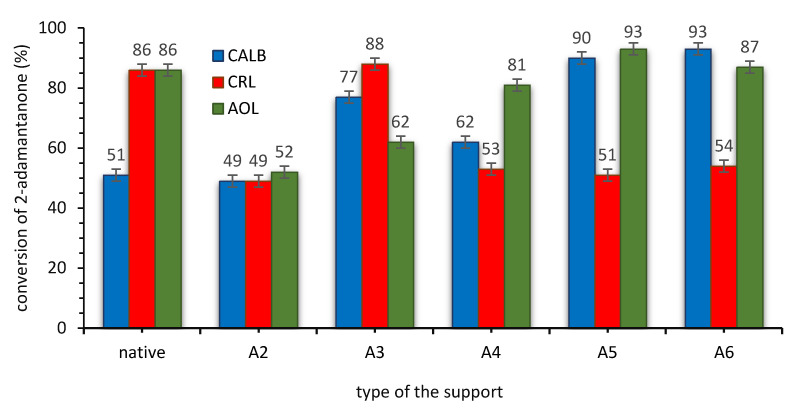
The activity of SILLP biocatalysts based on CNTs-CONH core in the Baeyer–Villiger oxidation of 2-adamantanone. Reaction conditions: 2-adamantanone 1 mmol (0.150 g), toluene 0.5 mL, *n*-octanoic acid 0.5 mL, 1 g of native lipase or 0.080 g CNTs-CONH-based biocatalyst, 35% aq. H_2_O_2_ 2 mmol (0.194 g), 20 °C, 4 h, 400 rpm.

**Figure 2:**
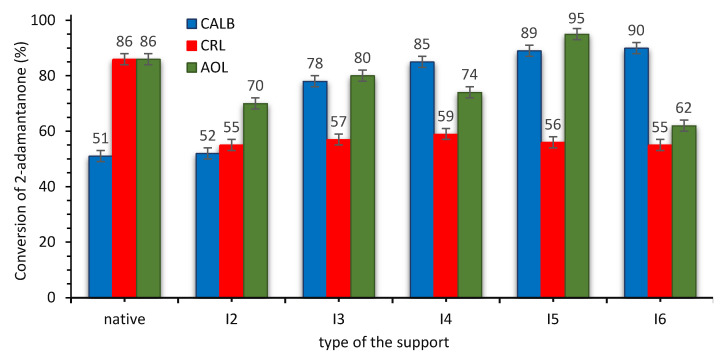
The activity of SILLP biocatalysts based on CNTs-C=N core in the Baeyer–Villiger oxidation of 2-adamantanone. Reaction conditions: 2-adamantanone 1 mmol (0.150 g), toluene 0.5 mL, *n*-octanoic acid 0.5 mL, 1 g of native lipase or 0.080 g CNTs-C=N-based biocatalyst, 35% aq. H_2_O_2_ 2 mmol (0.194 g), 20 °C, 4 h, 400 rpm.

The synthesized biocatalysts based on CNTs-CONH and CNTs-C=N-based cores were tested in the chemo-enzymatic Baeyer–Villiger oxidation of 2-adamantanone (Figure 1 and Figure 2). A structure of the anion significantly influenced the further stabilization of the protein. Hence, ILs with a diversity of anions was compared. In the synthesized SILLP biocatalysts, the linking bond can influence the activity and stability of an enzyme as well. These factors were compared, and the most active and stable biocatalyst was selected consequently.

Basically, physicochemical properties of the ionic liquid (i.e., hydrophobicity/hydrophilicity, polarity, nucleophilicity of anions, the tendency to form hydrogen bonds, viscosity, density, enzyme dissolution, or the surfactant effect) strongly influenced stability of the enzymes. Selection of the main factor responsible for the stabilizing properties emerged as practically impossible; hence, we designed the SILLP biocatalysts with a similar cation anchored to the support and various anions. This approach allowed to eliminate the role of physical properties of an IL from the enzyme-IL interactions. Thus, chemical factors were dominant; in addition, the activity of a lipase varied and depended on the type of the support as well. These factors allowed to analyze the influence of chemical structure of ionic species on further activity of the enzyme [21,22,23,24,25,31,32].

The studies exhibited the lowest activity of CRL in the model oxidation od 2-adamantanone—in comparison with CALB and AOL in almost all presented cases (excluding structure A3). The activity of SILLP biocatalysts of structures A2 and I2 (after quaternization with 1-hexyl iodide) based on I^-^ anions were rather poor in both cases (up to 70% after 4 h); hence, further improvement in this approach was required. Ion exchange reactions with hydrophobic anions [N(Tf)_2_]^-^ (structures A3 and I3) and [N(CN)_2_]^-^ (structures A4 and I4) allowed to obtain higher conversion of the ketone (up to 88% after 4 h). Subsequently, alkylations with dioctyl sulfate and trioctyl phosphate were performed to obtain structures A5, A6, I5, and I6 with hydrophilic anions. This approach allowed to obtain the highest conversion of the model ketone (up to 95% after 4 h). The Hofmeister effect is a phenomenon important in case of higher concentrations of ions observed in a micro-scale between solid/liquid phases in a reaction system. Hence, the Hofmeister series, which presents a tendency of anions to stabilize the proteins, clearly explained the stabilizing effect of octyl sulfate and dioctyl phosphate anions. Relatively chaotrophic cation anchored in the structure fits well to the application of the most kosmotrophic anions and highly active biocatalysts could have been synthesized as a result. Hence, the hydrophobicity/hydrophilicity of anions has a weaker impact on the stabilization of a protein after immobilization of the IL covalently on the solid support than in a solution, or in the case of physical immobilization of ILs [22,55,56].

The activity of SILLP biocatalysts based and CNTs-C=N core is generally lower. Only biocatalysts based on [Oc_2_PO_4_]^−^ anion and AOL enzyme were highly active (over 90% of the lactone yield after 4 h of the reaction). Nevertheless, recyclability attempts eliminated applicability of AOL as an active phase due to the low stability of the biocatalyst (ESI—Section S8). In the case of AOL, conversion of 2-adamantanone in the second reaction runs usually dropped over 20–30%, in contrast to the unchanging activity of CALB. Thus, CALB was selected to the further studies using biocatalyst with CNTs-CONH cored and [Oc_2_PO_4_]^−^ anion in the structure of the support.

For the comparison, pristine (unmodified) MWCNTs were used for the adsorption of enzymes (CALB content 15.7 wt.%) and this biocatalyst was tested in Baeyer–Villiger oxidation in the same reaction conditions, using 0.032 g (the same amount of an active phase than in the most active SILLP biocatalyst) of biocatalyst for 1 mmol of ketone. The activity of the resulted biocatalysts was lower (conversion of 2-adamantanone was 44%) what is an evident proof on the ILs activation role on the enzyme in this process.

#### 3.2.2. Influence of the Amount of SILLP Biocatalyst

**Figure 3:**
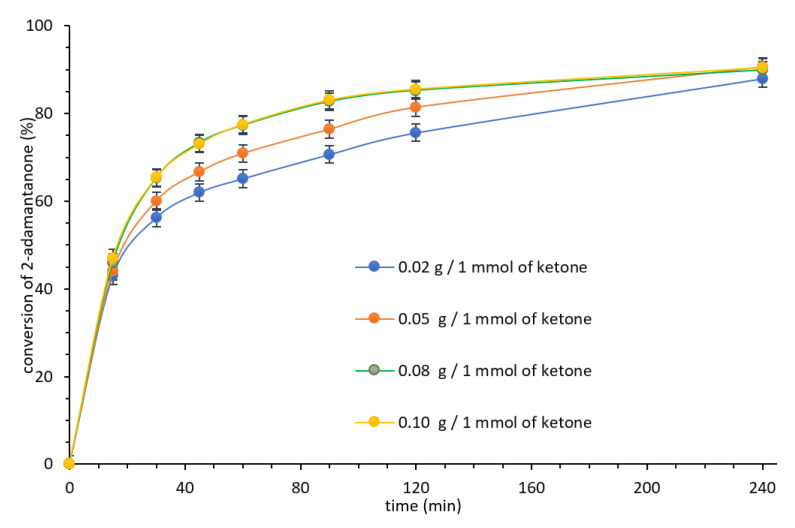
Influence of the amount of CALB-CNTs-CONH-Oc_2_PO_4_ biocatalyst on the Baeyer–Villiger oxidation rate. Reaction conditions: 2-adamantanone 1 mmol (0.150 g), toluene 0.5 mL, *n*-octanoic acid 0.5 mL, various amount of CALB-CNTs-CONH-Oc_2_PO_4_ biocatalyst, 35% aq. H_2_O_2_ 2 mmol (0.194 g), 20 °C, 400 rpm.

In the next step, an influence of the amount of biocatalyst was determined. Thus, experiments in a presence of 0.02–0.10 g of CALB-CNTs-CONH-Oc_2_PO_4_ biocatalyst were performed (Figure 3). The highest reaction rate was observed for 0.080 g of biocatalyst per 1 mmol of 2-adamantanone, while its higher amount 0.100 g did not influence any longer on the reaction rate; hence, it emerged that previously sufficient amount of CALB was introduced to the process with a biocatalyst.

#### 3.2.3. Stability of SILLP Biocatalyst

**Figure 4:**
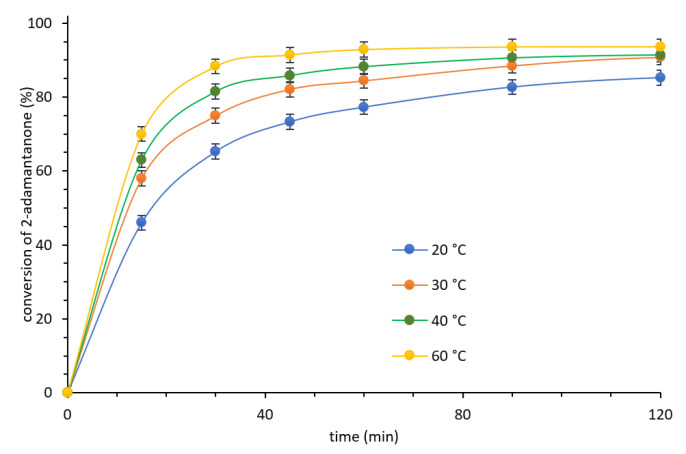
Influence of the temperature on the Baeyer–Villiger oxidation rate. Reaction conditions: 2-adamantanone 1 mmol (0.150 g), toluene 0.5 mL, *n*-octanoic acid 0.5 mL, 0.080 g CNTs-CONH- Oc_2_PO_4_ biocatalyst, 35% aq. H_2_O_2_ 2 mmol (0.194 g), various temperature, 400 rpm.

**Figure 5:**
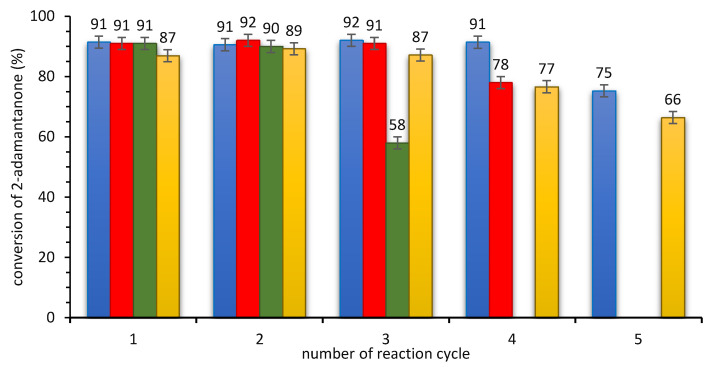
Recycling of the SILLP biocatalysts: CNTs-CONH-Oc_2_PO_4_ (A5) at 20 °C (blue), CNTs-CONH-Oc_2_PO_4_ (A5) at 40 °C (red), CNTs-CONH-Oc_2_PO_4_ (A5) at 60 °C (green), and CNTs-C=N-Oc_2_PO_4_ (I5) at 20 °C (yellow). Reaction conditions: 2-adamantanone 1 mmol (0.150 g), toluene 0.5 mL, *n*-octanoic acid 0.5 mL, 0.080 g of biocatalyst, 35% aq. H_2_O_2_ 2 mmol (0.194 g), 20–60 °C, 240 min at 20 °C; 90 min at 40 °C or 45 min at 60 °C, 400 rpm.

Next, the influence of the temperature (20–60 °C) on the stability of the CALB-CNTs-CONH-Oc_2_PO_4_ biocatalyst was also determined (Figure 4 and Figure 5).

These experiments showed that, due to kinetic issues, increasing temperature positively affected the total reaction rate and allowed to reduce the reaction times. Nevertheless, higher temperature led to a drop in the stability of biocatalytic systems (four reaction cycles at 20 °C, three reaction cycles at 40 °C, and two reaction cycles at 60 °C, without drop in activity). In addition, the stability of CNTs-CONH-Oc_2_PO_4_ biocatalyst was compared with stability of CALB-CNTs-C=N-Oc_2_PO_4_. The tendency of CALB to promiscuous hydrolysis of amine bond was suspected as the reason of decrease of CALB activity in the further reaction cycles. However, the imine bond did not improve the stability of the further biocatalyst. Hence, the Lowry’s protein detection method and TG analysis of biocatalysts after the last reaction cycle (ESI—Section S4) were applied to determine the amount of CALB in the reaction mixture. These studies confirmed lack of the leaching of the lipase to the reaction system. In addition, harsh conditions of the so-involved hydrogen peroxide affected negatively on the activity of CALB with possible degradation of its structure. Moreover, high amount of water or another hydrophilic compound can decrease the activity of a lipase in the reaction due to formation of an aqueous layer between the lipase and support [7,12].

**Figure 6:**
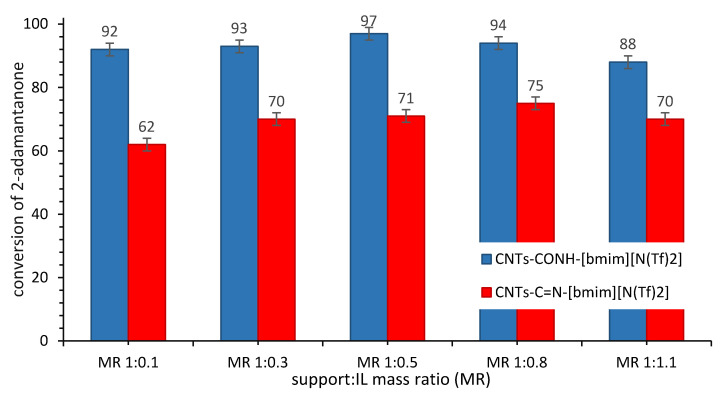
Influence of the CNTs-CONH/CNTs-C=N to [bmim][N(Tf)_2_] mass ratio (MR) on further activity of biocatalysts. Reaction conditions: 2-adamantanone 1 mmol (0.150 g), toluene 0.5 mL, *n*-octanoic acid 0.5 mL, 0.080 g CALB-CNTs-CONH-[bmim][N(Tf)_2_] biocatalyst with various MR or CALB-CNTs-C=N-[bmim][N(Tf)_2_] biocatalyst with various MR, 35% aq. H_2_O_2_ 2 mmol (0.194 g), 20 °C, 60 min, 400 rpm.

For comparative studies, the catalytic performance of SILP biocatalysts obtained via physical adsorption of [bmim][N(Tf)_2_] on the surface of CNTs-CONH and CNTs-C=N was studied. The activity and stability of [bmim][N(Tf)_2_] anchored to the CNTs-CONH (CALB-CNTs-CONH-[bmim][N(Tf)_2_] and on the CNTs-C=N (CALB-CNTs-C=N-[bmim][N(Tf)_2_] was compared with their covalently bonded counterparts. First, the influence of the mass ratio (MR) of CNTs-CONH/CNTs-C=N to [bmim][N(Tf)_2_] on the final biocatalyst activity was determined. The most active were biocatalysts obtained of the MR 1:0.5–1:0.8 (Figure 6); nevertheless, application of CALB-CNTs-CONH-[bmim][N(Tf)_2_] allowed to obtain higher conversion of the substrate (99% in 4 h in comparison with 88% in 4 h in case of application CALB-CNTs-C=N-[bmim][N(Tf)_2_]). Table 2 presents a loading of IL and CALB in each case, hence the possible reason for dropping in CALB’s activity in case of using higher amount of IL at the synthesis step is increasing of an amount of adsorbed CALB as well, which leads to formation of its dimeric agglomerates [12]. [bmim][N(Tf)_2_]) was selected due to the lower activity of CALB in case of application of [bmim][Oc_2_PO_4_] for the synthesis of SILP biocatalysts (in favorable conditions: 79% conversion of 2-adamantanone in case of application of CNTs-CONH support and 65% conversion of 2-adamantanone if CNTs-C=N support was applied instead). This behavior clearly confirmed that physical immobilization of IL continued from the solution, and other factors, i.e., hydrophobicity/hydrophilicity, density, or viscosity, are responsible for the stabilizing effects, otherwise the results would have been similar to those with application of SILLP biocatalysts.

**Figure 7:**
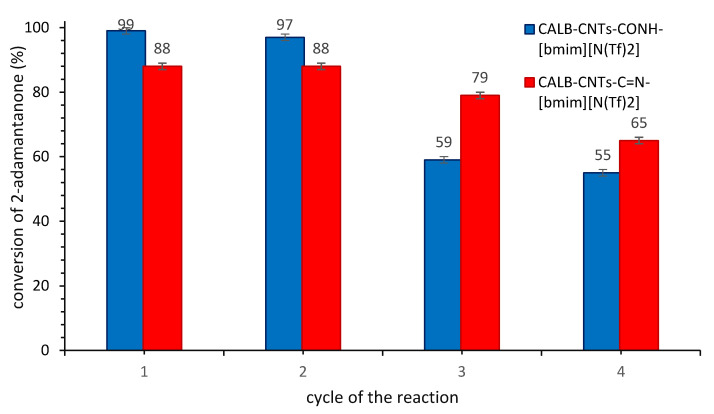
Recycle of the SILP biocatalyst. Reaction conditions: 2-adamantanone 1 mmol (0.150 g), toluene 0.5 mL, *n*-octanoic acid 0.5 mL, 0.080 g CALB-CNTs-CONH-[bmim][N(Tf)_2_] biocatalyst with MR support: Il 1:0.5 or CALB-CNTs-C=N-[bmim][N(Tf)_2_] biocatalyst with MR support: Il 1:0.8, 35% aq. H_2_O_2_ 2 mmol (0.194 g), 20 °C, 4 h, 400 rpm.

Next, the stability of both the most promising biocatalysts was determined (Figure 7). To this aim, four subsequent reaction cycles were carried out. The evident drop in the activity of both biocatalysts was observed after the second cycle. The reason could be the leaching of the IL from the surface of the support, chemical deactivation of an enzyme or an accommodation of water molecules or another hydrophilic compounds [18,19,20]. Lowry’s protein detection technique confirmed lack of the lipase in filtrate. In addition, to determine what amount of IL and CALB remained on the support, thermogravimetric analysis was performed. These studies confirmed that the most possible reason for the drop in activity of the SILP biocatalyst was the chemical deactivation or adsorption of another hydrophilic compound, due to an increase in the mass loss a range comparable with the fresh biocatalysts. Application of CALB-CNTs-C=N-[bmim][N(Tf)_2_]) allowed to maintain a higher activity of an enzyme for longer. Thus, the application of the SILP approach did not affect positively on the lengthen lifetime of CALB in comparison with presented before (Figure 5) SILLPs structures.

## 4. Conclusions

We have presented highly stable and active lipase-based biocatalysts dedicated to chemo-enzymatic Baeyer–Villiger oxidation of 2-adamantanone. This approach allowed to decrease an amount of CALB in the biocatalyst in comparison with the CALB immobilized via interfacial activation on pristine, unmodified multi-walled carbon nanotubes. In addition, improvement in a thermal stability was demonstrated. It is worth to emphasize that our work presents the first successful attempt on the implementation of supported ionic liquid phase technique in the extremely aggressive environment of hydrogen peroxide. Application of supported ionic liquid-like phase in order to anchoring ionic liquids to the support allowed the elimination of physical factors from the IL-enzyme interactions at a molecular level, hence the influence of the structures of anchoring ions on the stability of proteins was much more predictable. In addition, covalent modification of the surface of multi-walled carbon nanotubes was much effective than physical adsorption of IL towards the synthesis of CNTs-based SILP biocatalysts.

## Figures and Tables

**Table 1:** Characterization of supports and biocatalysts used in this study.

Material	Content of Functional Groups (wt.%) *	Content of CALB (wt.%) *	Content of CRL (wt.%) *	Content of AOL (wt.%) *
CNTs-COOH	1.4	N/A	N/A	N/A
CNTs-CONH (A1)	5.8	N/A	N/A	N/A
CNTs-CONH-HEX-I (A2)	6.4	2.7	7.9	5.0
CNTs-CONH-HEX-N(Tf)_2_ (A3)	9.1	2.8	1.1	6.7
CNTs-CONH-HEX-N(CN)_2_ (A4)	7.0	2.3	4.7	5.3
CNTs-CONH-Oc_2_PO_4_ (A5)	10.9	6.4	1.2	1.9
CNTs-CONH-OcSO_4_ (A6)	8.3	4.1	6.5	1.6
CNTs-CHO	0.7	N/A	N/A	N/A
CNTs-C=N (I1)	3.2	N/A	N/A	N/A
CNTs-C=N-HEX-I (I2)	4.3	5.9	3.4	7.8
CNTs-C=N-HEX-N(Tf)_2_ (I3)	7.5	0.6	9.2	4.1
CNTs-C=N-HEX-N(CN)_2_ (I4)	5.1	1.9	5.3	3.7
CNTs-C=N-Oc_2_PO_4_ (I5)	2.7	3.1	7.1	2.6
CNTs-C=N-OcSO_4_ (I6)	2.6	2.6	6.2	5.0

* calculated using thermogravimetric analysis.

**Table 2:** Characterization of supports and biocatalysts used in this study.

Material	[bmim][N(Tf)_2_] Content (wt.%)	CALB Content (wt.%)
CNTs-CONH-[bmim][NTf_2_] (MR 1:0.1)	0.4	2.8
CNTs-CONH-[bmim][NTf_2_] (MR 1:0.3)	1.2	4.5
CNTs-CONH-[bmim][NTf_2_] (MR 1:0.5)	2.2	8.0
CNTs-CONH-[bmim][NTf_2_] (MR 1:0.8)	3.2	18.6
CNTs-CONH-[bmim][NTf_2_] (MR 1:1.1)	3.4	19.2
CNTs-C=N-[bmim][NTf_2_] (MR 1:0.1)	0.1	5.1
CNTs-C=N-[bmim][NTf_2_] (MR 1:0.3)	0.7	5.7
CNTs-C=N-[bmim][NTf2] (MR 1:0.5)	1.1	6.7
CNTs-C=N-[bmim][NTf_2_] (MR 1:0.8)	2.2	15.6
CNTs-C=N-[bmim][NTf_2_] (MR 1:1.1)	2.8	17.0

## Data Availability

Data sharing is not applicable for this article.

## Supplementary Materials

The following are available online at https://www.mdpi.com/article/10.3390/ma14133443/s1, Section S1: GC-MS analysis; Section S2: NMR analysis; Section S3: SEM-EDS analysis; Section S4: Thermogravimetric analysis; Section S5: IR analysis; Section S6: XPS analysis; Section S7: Elemental analysis; Section S8: Influence of the type of an enzyme on the recyclability of biocatalysts.
