# Supplementary material for: Chemo-Enzymatic Baeyer–Villiger Oxidation Facilitated with Lipases Immobilized in the Supported Ionic Liquid Phase
Source: Materials (Basel). 2021 Jun 22;14(13):3443. doi: 10.3390/ma14133443 (PMC8269471; doi:10.3390/ma14133443)
Supplement: Supplementary file 1 [file materials-14-03443-s001.zip › materials-1278377-supplementary.pdf]

Chemo-enzymatic Baeyer-Villiger oxidation facilitated with lipases immobilized in the supported ionic liquid phase

Anna Szelwicka [1], Anna Wolny [1], Miroslawa Grymel [2], Sebastian Jurczyk [3], Slawomir Boncel [2] and Anna Chrobok [1,*]

[1] Department of Chemical Organic Technology and Petrochemistry, Silesian University of Technology, Faculty of Chemistry, Krzywoustego 4, 44-100 Gliwice, Poland; Anna.Szelwicka@polsl.pl (A.S.); Anna.Wolny@polsl.pl (A.W.)

[2] Department of Organic Chemistry, Bioorganic Chemistry and Biotechnology, Silesian University of Technology, Faculty of Chemistry Krzywoustego 4, 44-100 Gliwice, Poland; Miroslawa.Grymel@polsl.pl (M.G.); Slawomir.Boncel@polsl.pl (S.B.)

[3] Institute for Engineering of Polymer Materials and Dyes, Lukasiewicz Research Network, Sklodowskiej-Curie 55, 87-100 Torun, Poland; Sebastian.Jurczyk@impib.lukasiewicz.gov.pl (S.J.)

*Correspondence: Anna.Chrobok@polsl.pl (A.C.); Tel.: +48-32-237-2917

Keywords: supported ionic liquid phase, biocatalysis, lipase, chemo-enzymatic Baeyer-Villiger oxidation, heterogeneous catalysis, carbon nanotubes, immobilization

Content

Section S1. GC-MS analysis 3

Section S2. NMR analysis 4

Section S3. SEM-EDS analysis 5

Section S4. Thermogravimetric analysis 11

Section S5. IR analysis 21

Section S6. XPS analysis 23

Section S7. Elemental analysis. 26

Section S8. Influence of the type of an enzyme on the recyclability of biocatalysts 27

Section S1. GC-MS analysis

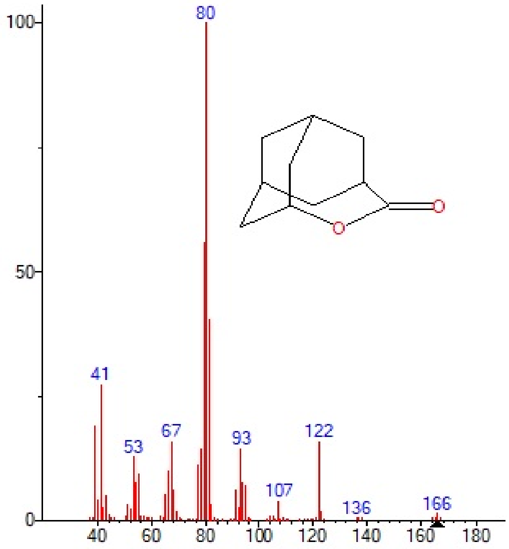

Figure S1. MS spectrum of 4-oxatricyclo[4.3.1.13.8]undecan-5-one (based on RTL mass spectral library).

Section S2. NMR analysis

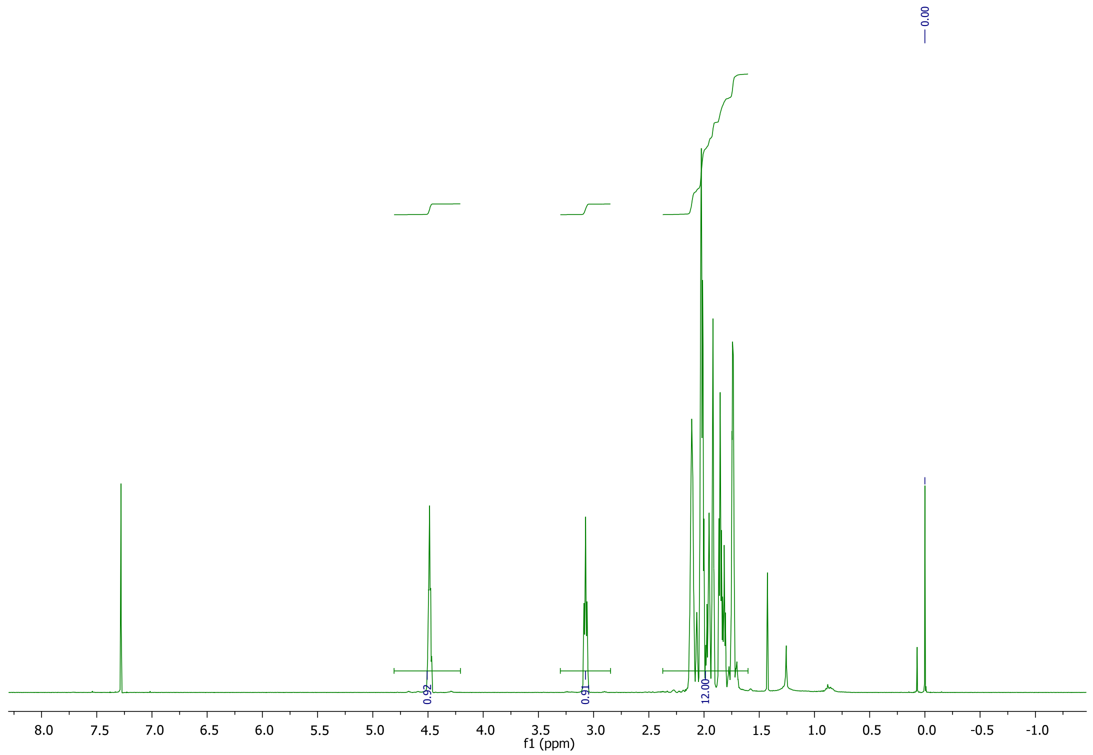

Figure S2. ^{1}H NMR spectrum of 4-oxatricyclo[4.3.1.13.8]undecan-5-one. 7.28 - $CHCL_3$ from $CDCL_3$. Impurities in a range of 0.0-1.5 ppm – solvents after extraction.

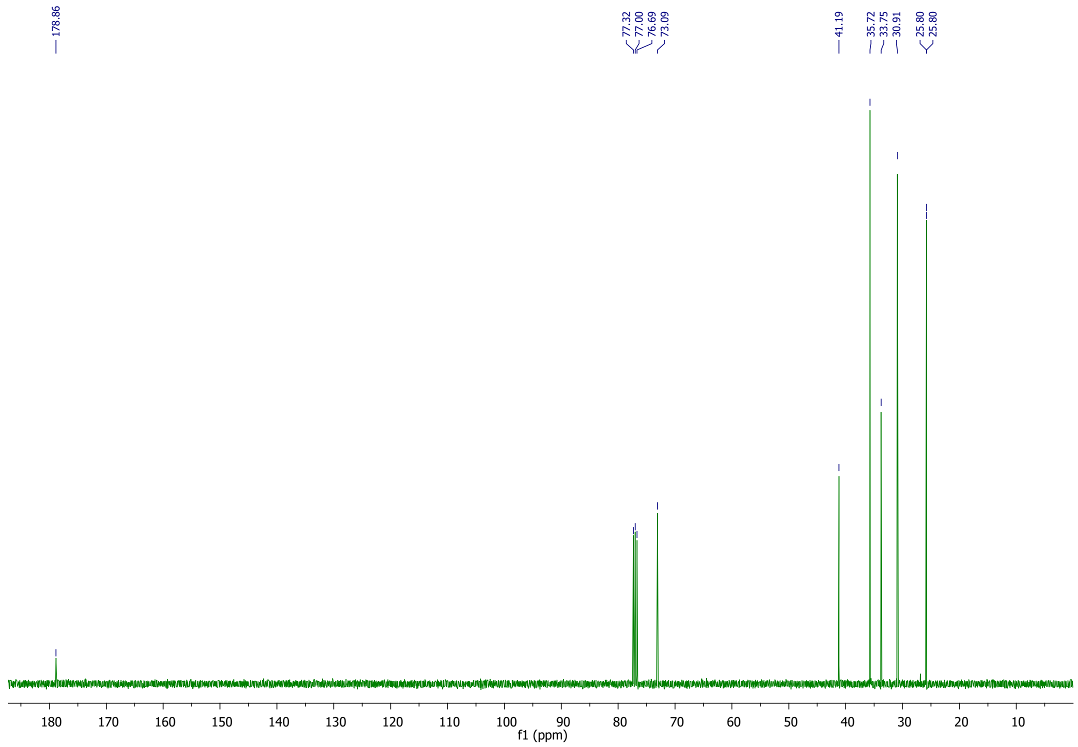

Figure S3. ^{13}C NMR spectrum of 4-oxatricyclo[4.3.1.13.8]undecan-5-one.

Section S3. SEM-EDS analysis

All SILLP materials were analyzed using SEM microscope equipped with EDS detector in order to confirm the presence of characteristic atoms in the structures. The qualitative analysis clearly confirmed a presence of expected elements in all structures.

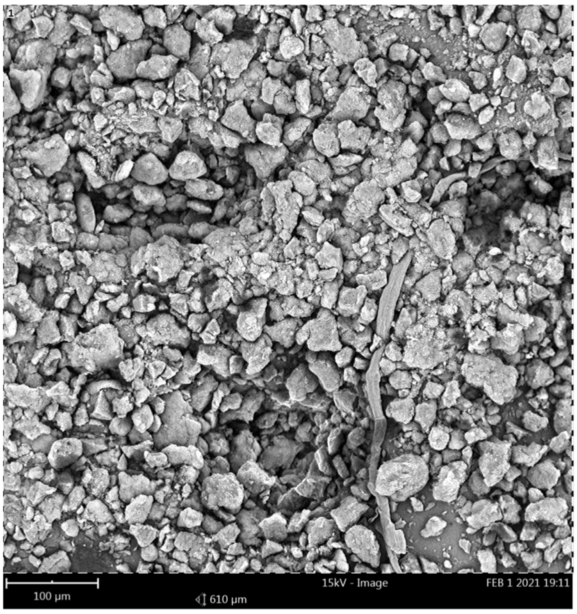

Element Number	Element Symbol	Element Name	Atomic Conc.	Weight Conc.
**6**	C	Carbon	88.38	85.81
**8**	O	Oxygen	6.38	8.25
**7**	N	Nitrogen	5.24	5.94

Figure S4. The SEM-EDS analysis of CNTs-CONH (A1).

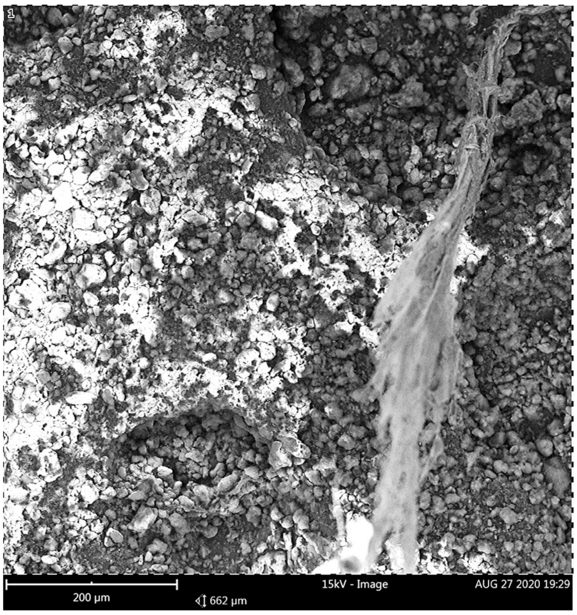

Element Number	Element Symbol	Element Name	Atomic Conc.	Weight Conc.
**6**	C	Carbon	83.72	79.17
**8**	O	Oxygen	9.89	12.46
**7**	N	Nitrogen	6.24	6.88
**53**	I	Iodine	0.15	1.49

Figure S5. The SEM-EDS analysis of CNTs-CONH-HEX-I (A2).

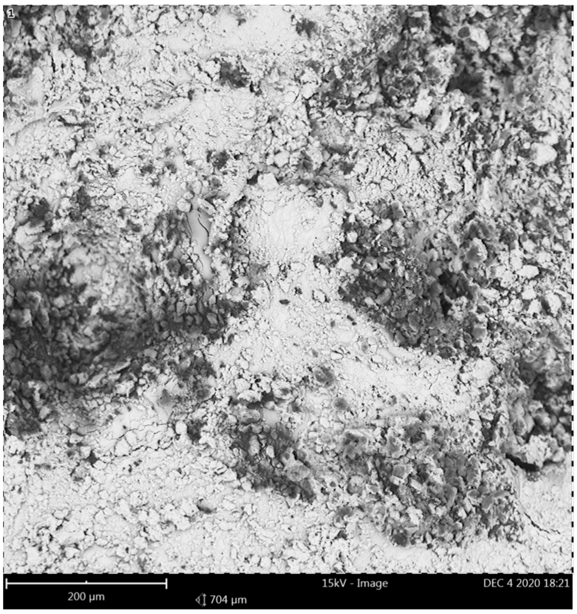

Element Number	Element Symbol	Element Name	Atomic Conc.	Weight Conc.
**6**	C	Carbon	76.65	73.03
**7**	N	Nitrogen	20.97	23.30
**9**	F	Fluorine	2.29	3.46
**16**	S	Sulfur	0.08	0.21

Figure S6. The SEM-EDS analysis of CNTs-CONH-HEX-N(Tf)$_2$ (A3).

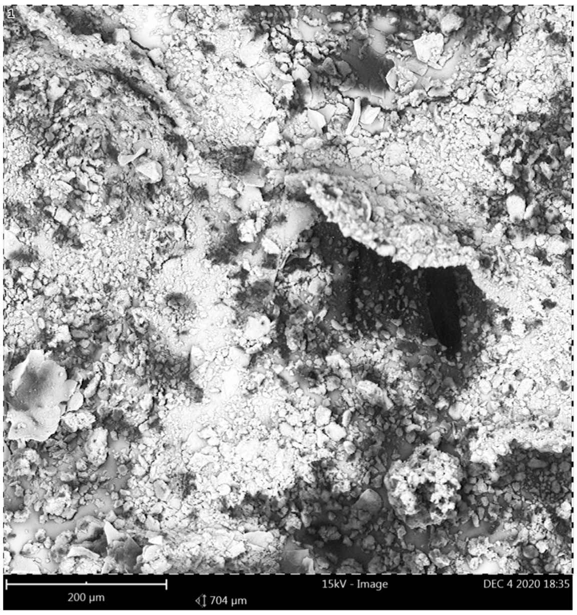

Element Number	Element Symbol	Element Name	Atomic Conc.	Weight Conc.
**6**	C	Carbon	74.54	71.52
**7**	N	Nitrogen	25.46	28.48

Figure S7. The SEM-EDS analysis of CNTs-CONH-HEX-N(CN)$_2$ (A4).

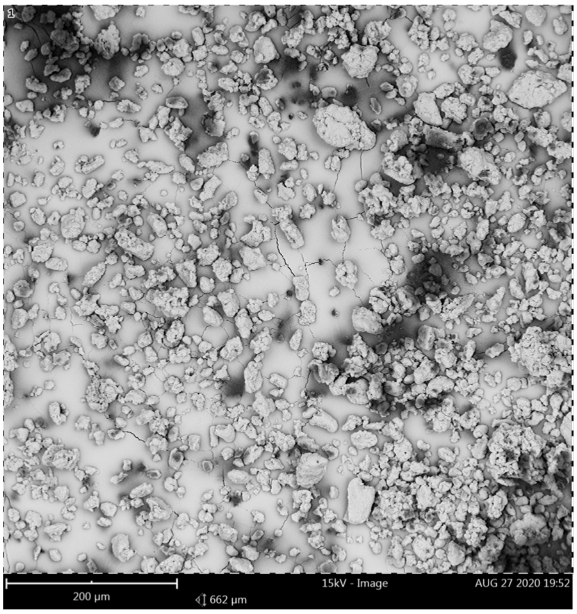

Element Number	Element Symbol	Element Name	Atomic Conc.	Weight Conc.
**6**	C	Carbon	57.80	52.28
**7**	N	Nitrogen	22.47	23.70
**8**	O	Oxygen	19.51	23.51
**15**	P	Phosphorus	0.22	0.51

Figure S8. The SEM-EDS analysis of CNTs-CONH-Oc$_2PO_4$ (A5).

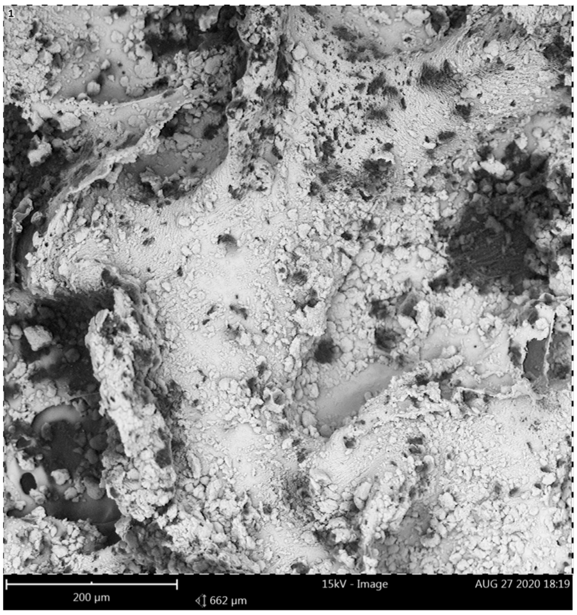

Element Number	Element Symbol	Element Name	Atomic Conc.	Weight Conc.
**6**	C	Carbon	56.50	50.42
**8**	O	Oxygen	28.41	33.78
**7**	N	Nitrogen	15.01	15.62
**16**	S	Sulfur	0.08	0.18

Figure S9. The SEM-EDS analysis of CNTs-CONH-OcSO_4 (A6).

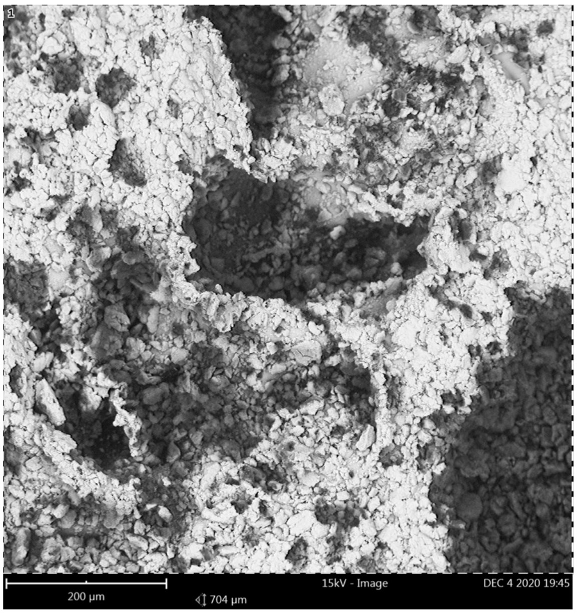

Element Number	Element Symbol	Element Name	Atomic Conc.	Weight Conc.
**6**	C	Carbon	84.10	80.49
**7**	N	Nitrogen	15.70	17.52
**53**	I	Iodine	0.20	1.98

Figure S10. The SEM-EDS analysis of CNTs-C=N-HEX-I (I2).

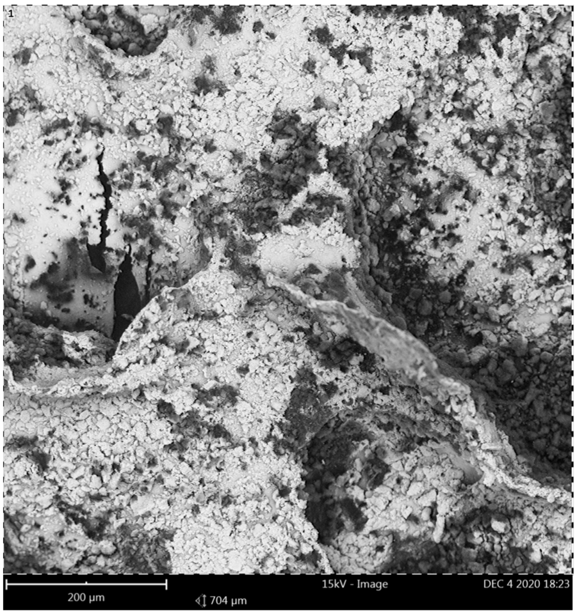

Element Number	Element Symbol	Element Name	Atomic Conc.	Weight Conc.
**6**	C	Carbon	78.23	74.65
**7**	N	Nitrogen	19.01	21.16
**9**	F	Fluorine	2.72	4.10
**16**	S	Sulfur	0.04	0.09

Figure S11. The SEM-EDS analysis of CNTs-C=N-HEX-N(Tf)$_2$ (I3).

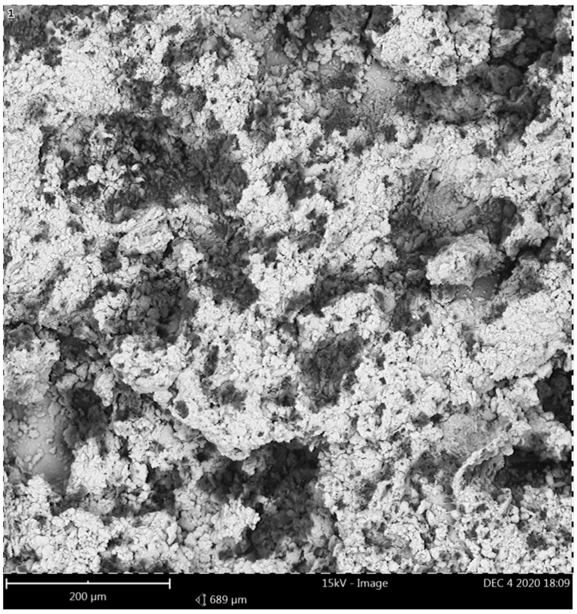

Element Number	Element Symbol	Element Name	Atomic Conc.	Weight Conc.
**6**	C	Carbon	74.10	71.04
**7**	N	Nitrogen	25.90	28.96

Figure S12. The SEM-EDS analysis of CNTs-C=N-HEX-N(CN)$_2$ (I4).

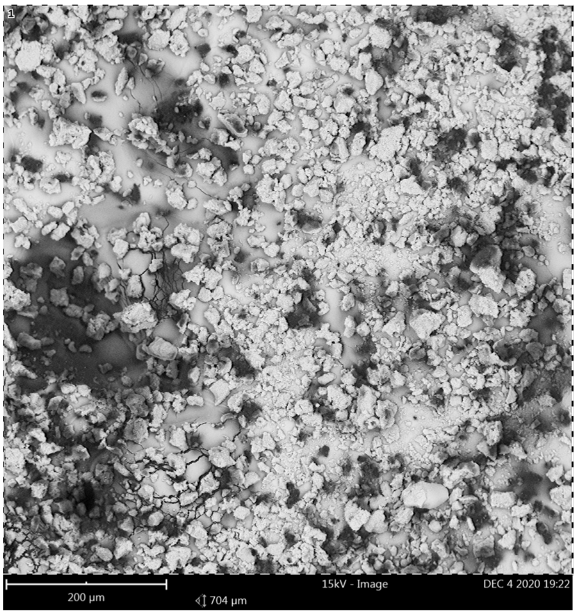

Element Number	Element Symbol	Element Name	Atomic Conc.	Weight Conc.
**6**	C	Carbon	72.65	67.59
**8**	O	Oxygen	16.83	20.86
**7**	N	Nitrogen	10.41	11.29
**15**	P	Phosphorus	0.11	0.26

Figure S13. The SEM-EDS analysis of CNTs-C=N-Oc$_2$PO$_4$ (I5).

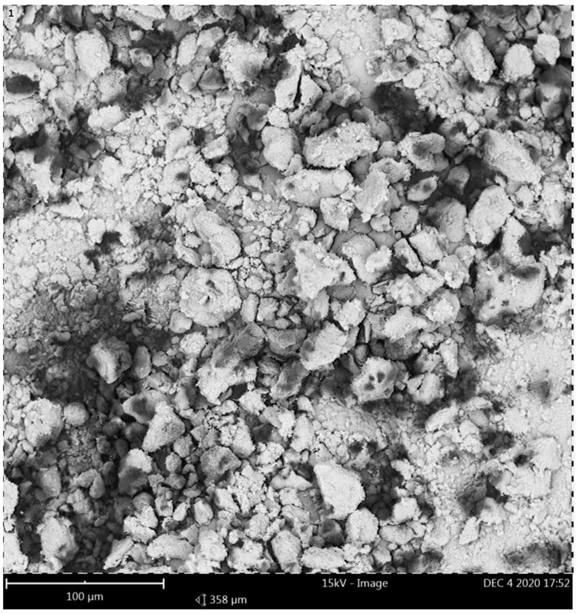

Element Number	Element Symbol	Element Name	Atomic Conc.	Weight Conc.
**6**	C	Carbon	69.20	63.86
**8**	O	Oxygen	15.21	18.69
**7**	N	Nitrogen	15.12	16.27
**16**	S	Sulfur	0.48	1.18

Figure S14. The SEM-EDS analysis of CNTs-C=N-$OcSO_4$ (I6).

Section S4. Thermogravimetric analysis

Thermograms were collected for all materials and biocatalysts. The content of functional groups or adsorbed IL was determined based on the mass loss during analysis. The lipase content was determined based on a difference between mass of a biocatalyst and a support in a range of 180-450 °C.

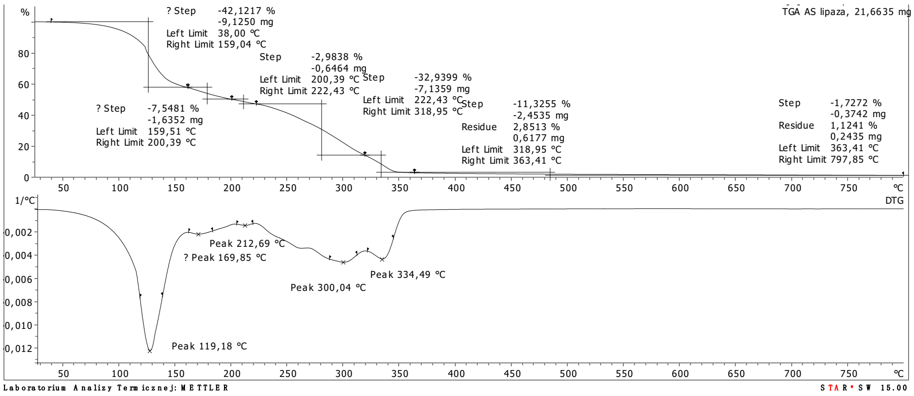

Figure S15. Thermogravimetric analysis of CALB.

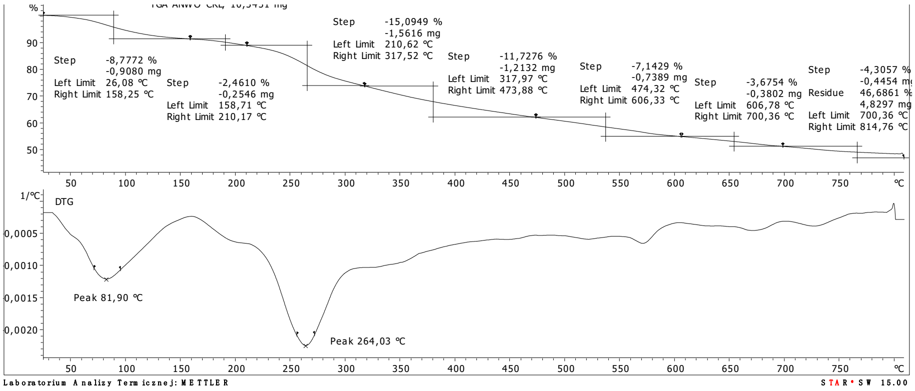

Figure S16. Thermogravimetric analysis of CRL.

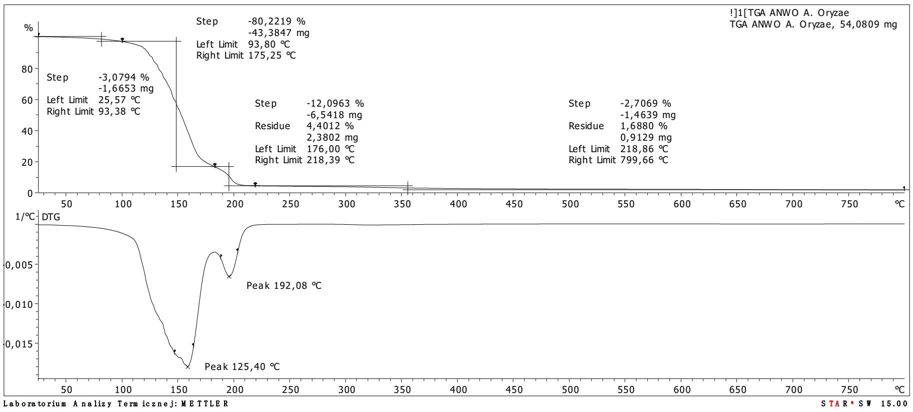

Figure S17. Thermogravimetric analysis of AOL.

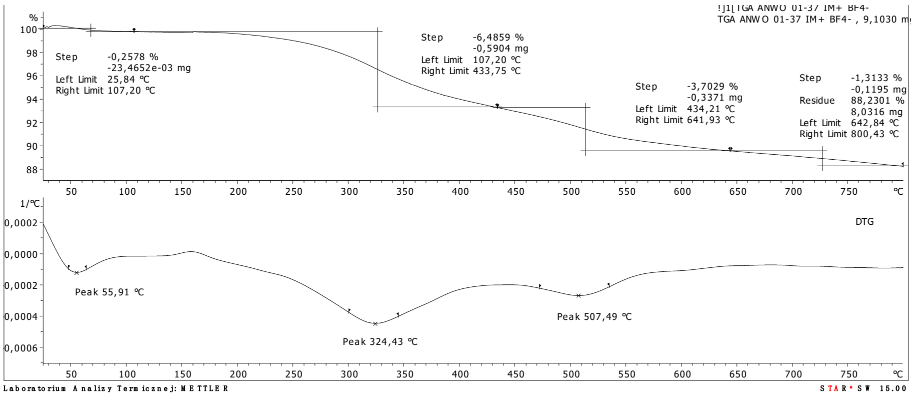

Figure S18. Thermogravimetric analysis of CNTs-CONH-Oc$_2$PO$_4$ (A5).

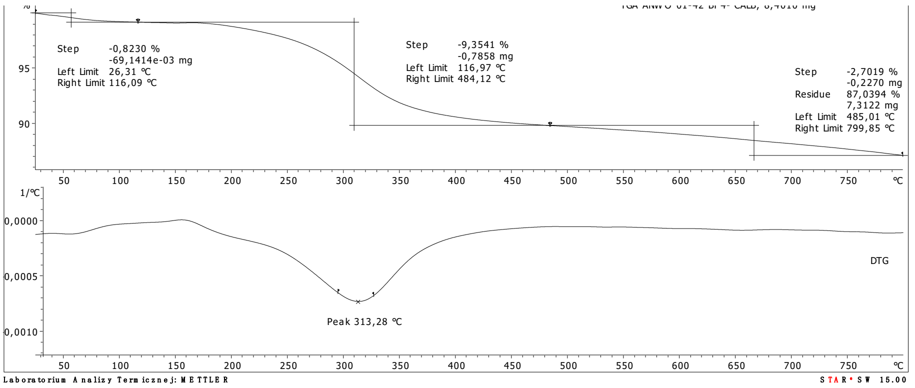

Figure S19. Thermogravimetric analysis of CALB-CNTs-CONH-Oc$_2$PO$_4$ (A5).

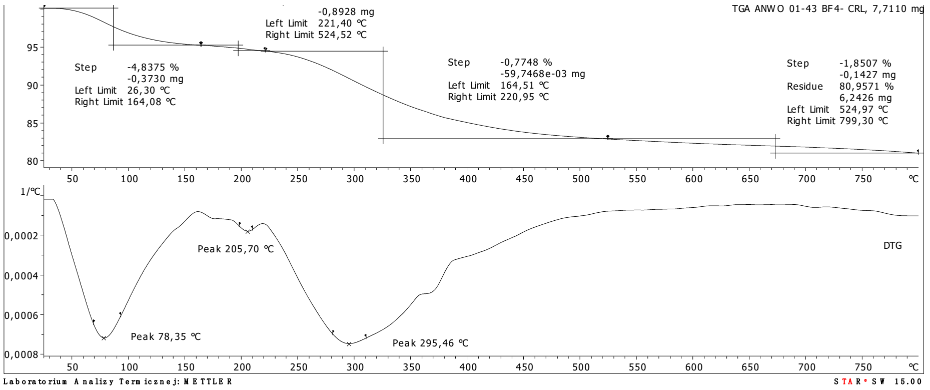

Figure S20. Thermogravimetric analysis of CRL-CNTs-CONH-Oc$_2$PO$_4$ (A5).

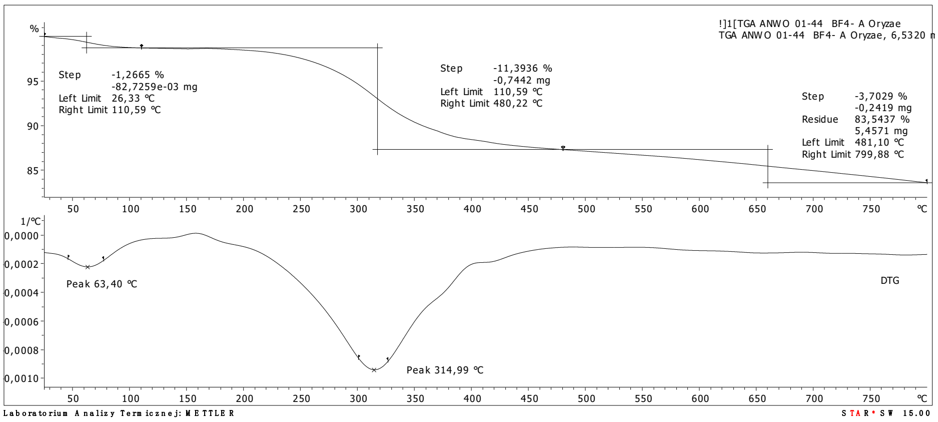

Figure S21. Thermogravimetric analysis of AOL-CNTs-CONH-Oc_2PO_4 (A5).

Analysis for the rest of covalent structures and biocatalysts was performed similarly, nevertheless due to size of data these analyses were not included in this SI. Analyses remain available upon request from authors.

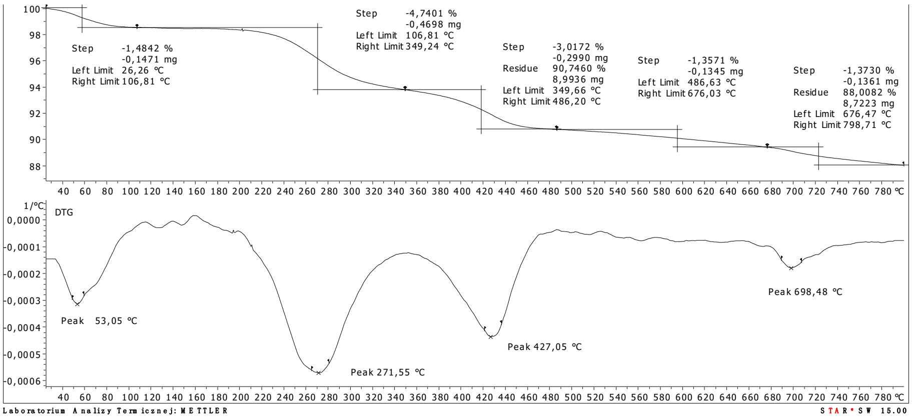

Figure S22. Thermogravimetric analysis of CNTs-CONH-[bmim][$N(Tf)_2$] MR 1:0.1

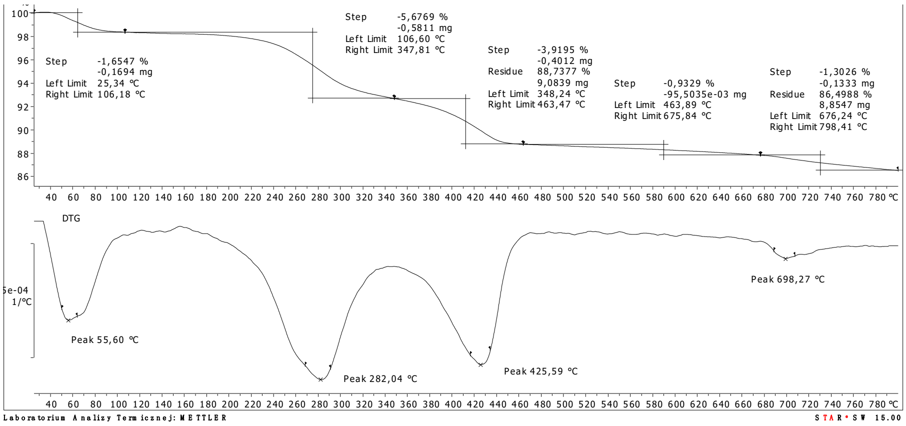

Figure S23. Thermogravimetric analysis of CNTs-CONH-[bmim][$N(Tf)_2$] MR 1:0.3

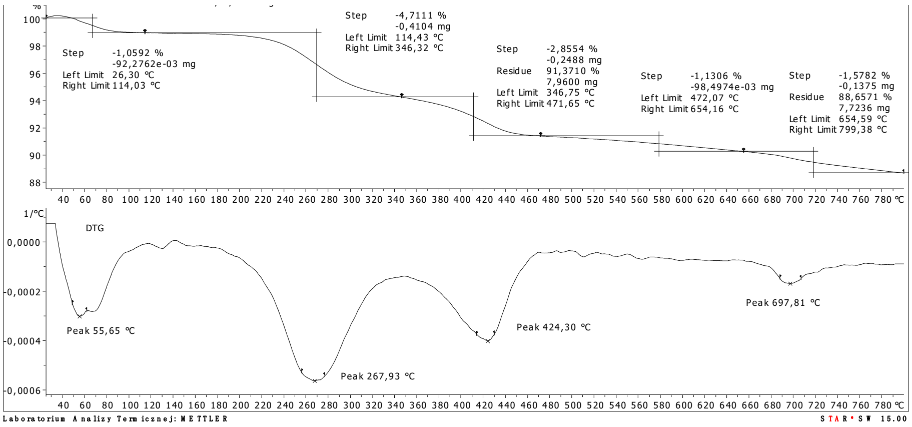

Figure S24. Thermogravimetric analysis of CNTs-CONH-[bmim][N(Tf)2] MR 1:0.5

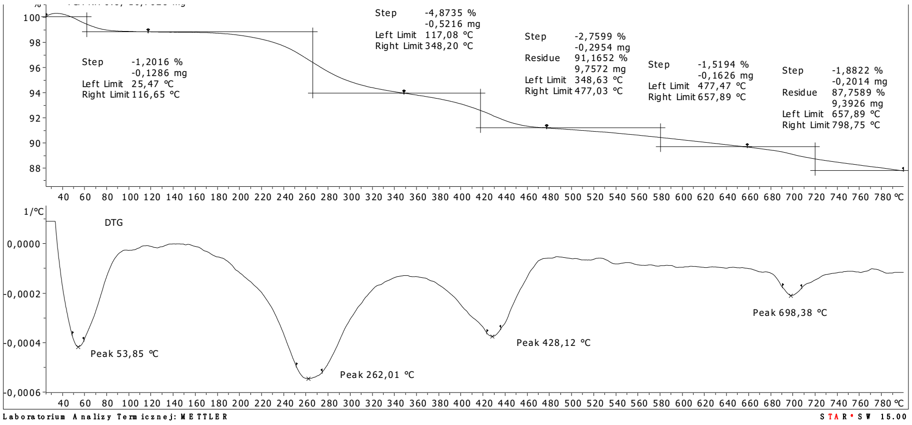

Figure S25. Thermogravimetric analysis of CNTs-CONH-[bmim][N(Tf)2] MR 1:0.8

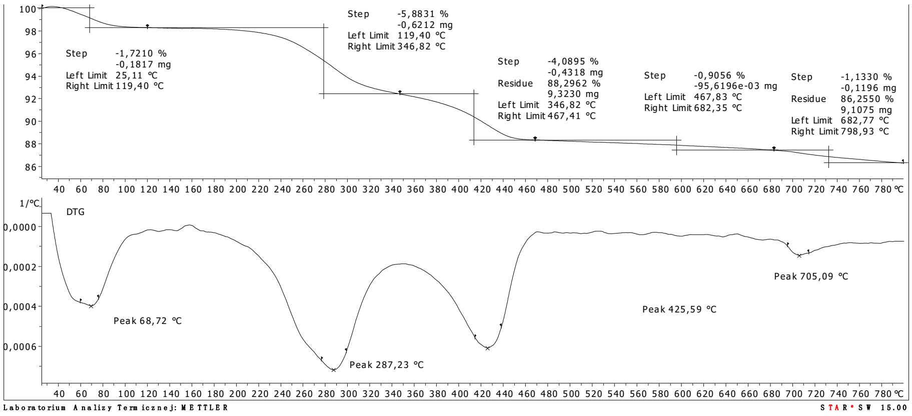

Figure S26. Thermogravimetric analysis of CNTs-CONH-[bmim][N(Tf)2] MR 1:1.1

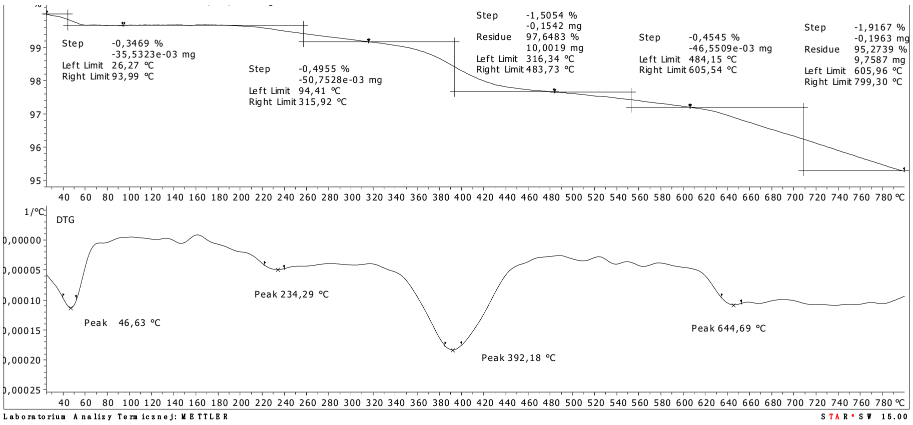

Figure S27. Thermogravimetric analysis of CNTs-C=N-[bmim][N(Tf)$_2$] MR 1:0.1

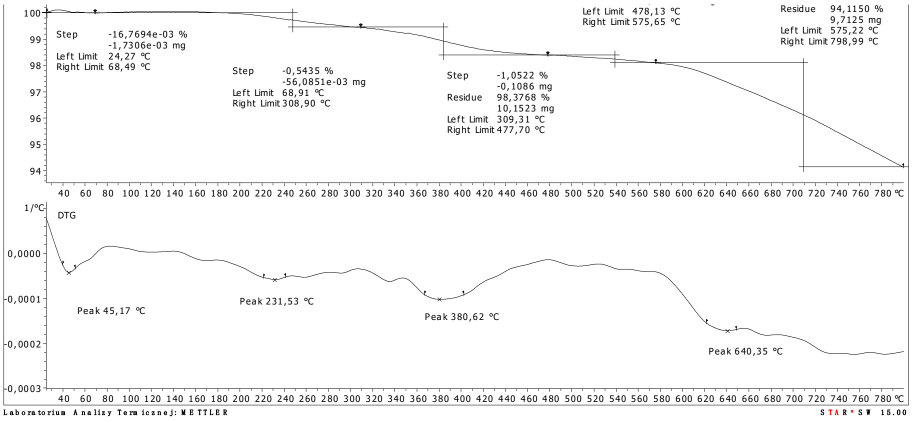

Figure S28. Thermogravimetric analysis of CNTs-C=N-[bmim][N(Tf)$_2$] MR 1:0.3

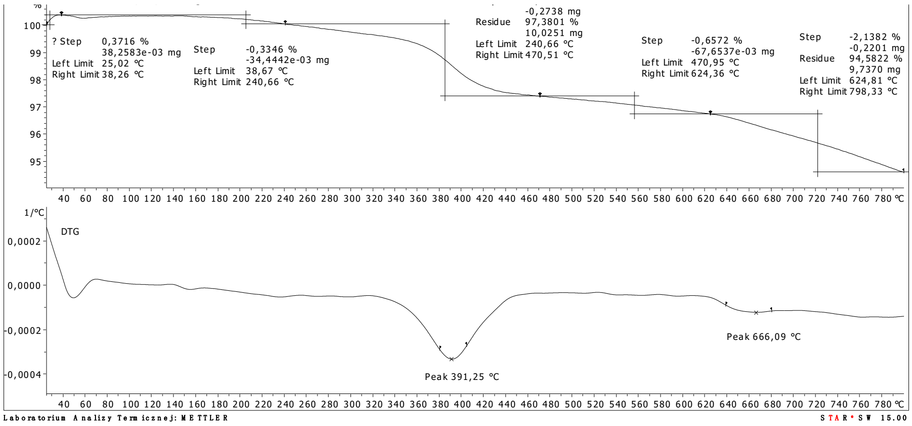

Figure S29. Thermogravimetric analysis of CNTs-C=N-[bmim][N(Tf)$_2$] MR 1:0.5

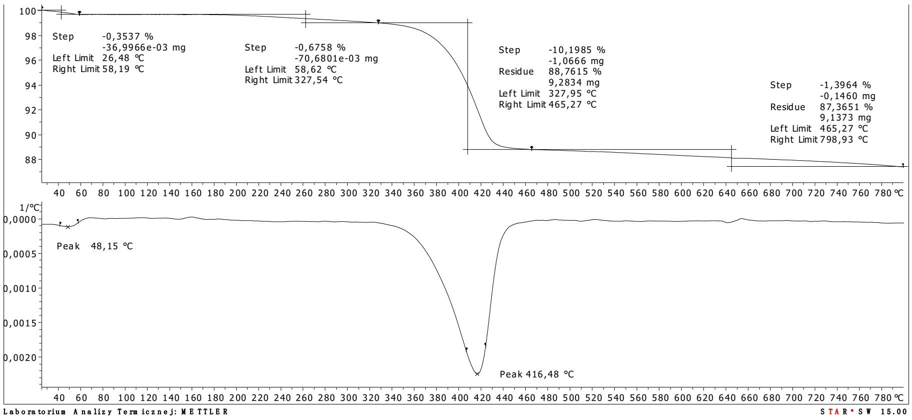

Figure S30. Thermogravimetric analysis of CNTs-C=N-[bmim][N(Tf)$_2$] MR 1:0.8

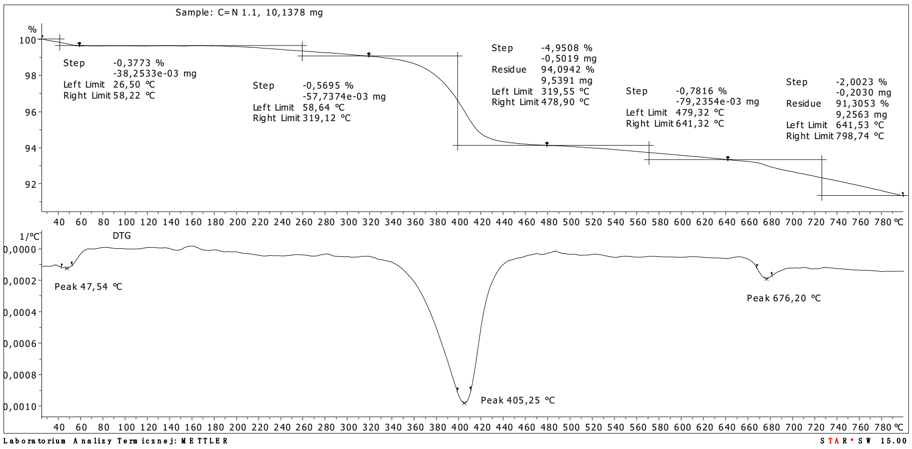

Figure S31. Thermogravimetric analysis of CNTs-C=N-[bmim][N(Tf)$_2$] MR 1:1.1

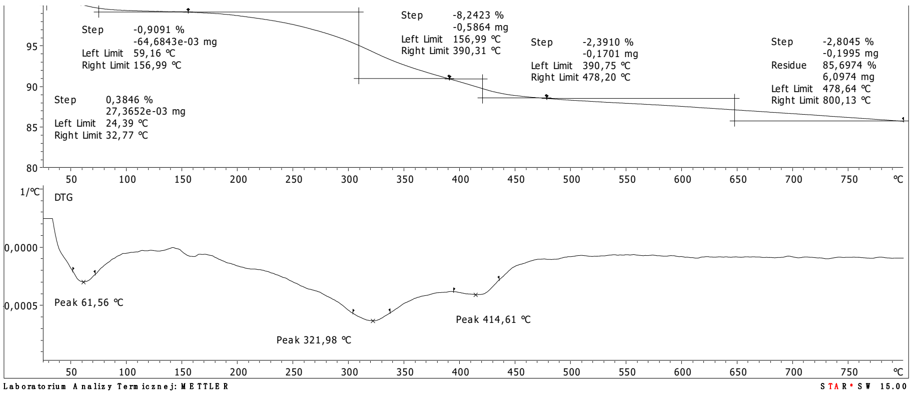

Figure S32. Thermogravimetric analysis of CALB-CNTs-CONH-[bmim][N(Tf)$_2$] MR 1:0.1

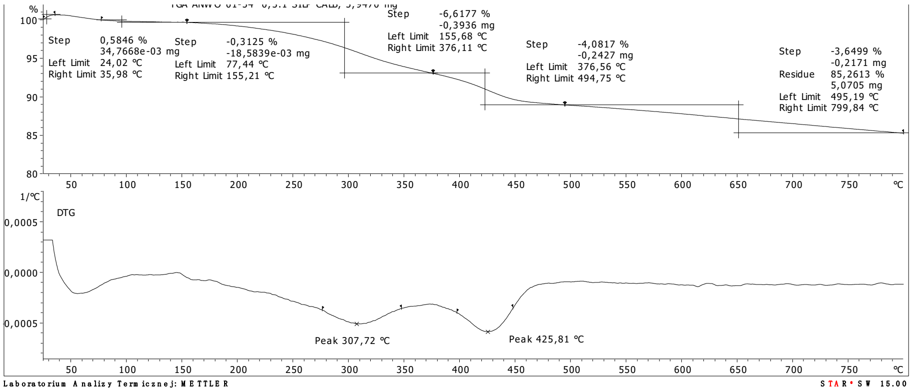

Figure S33. Thermogravimetric analysis of CALB-CNTs-CONH-[bmim][N(Tf)$_2$] MR 1:0.3

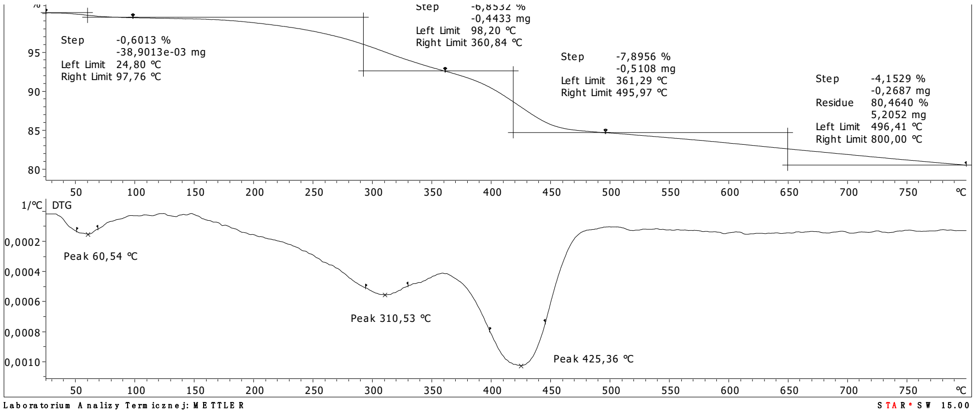

Figure S34. Thermogravimetric analysis of CALB-CNTs-CONH-[bmim][N(Tf)$_2$] MR 1:0.5

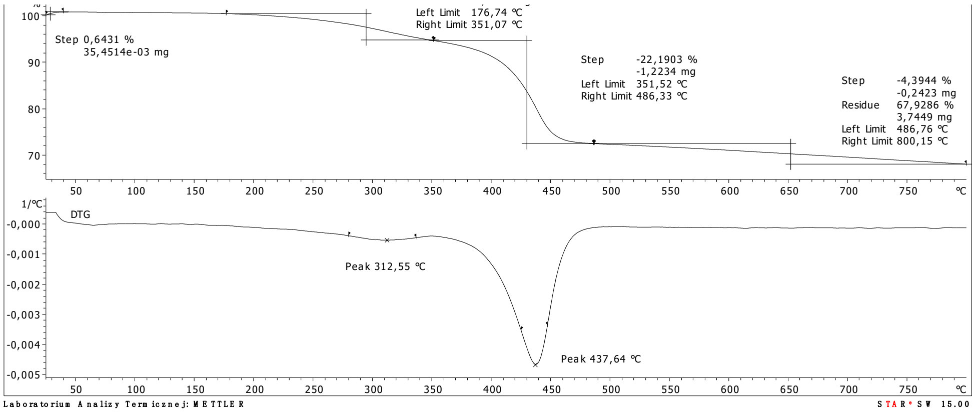

Figure S35. Thermogravimetric analysis of CALB-CNTs-CONH-[bmim][N(Tf)$_2$] MR 1:0.8

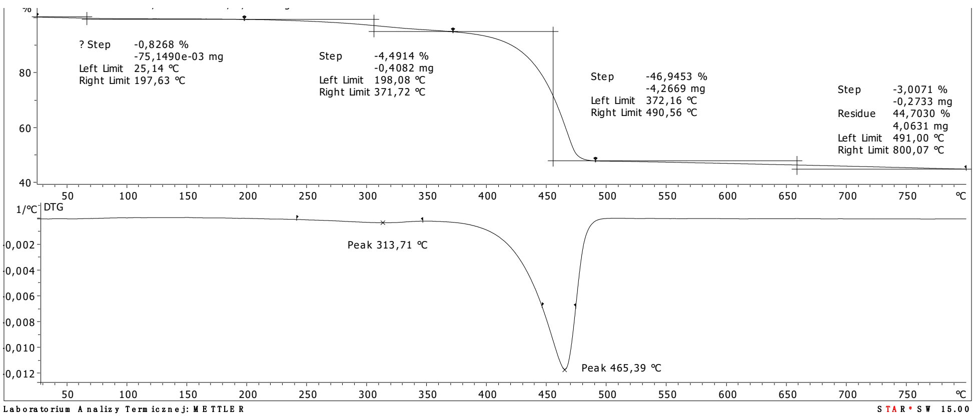

Figure S36. Thermogravimetric analysis of CALB-CNTs-CONH-[bmim][N(Tf)$_2$] MR 1:1.1

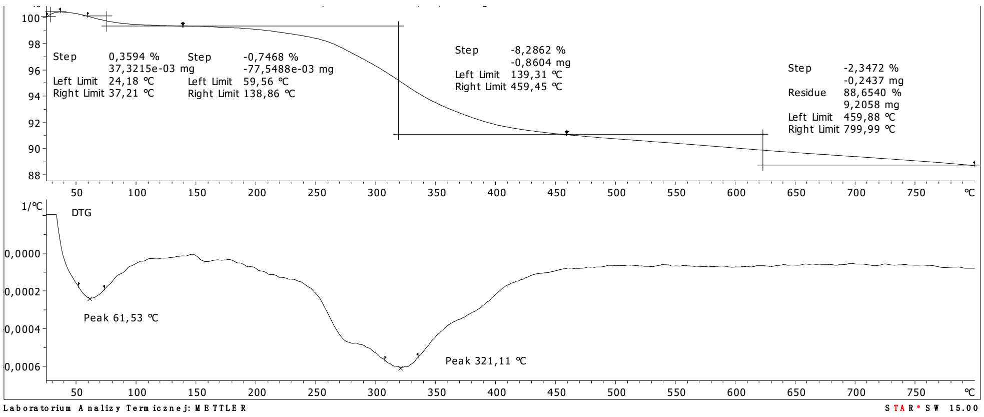

Figure S37. Thermogravimetric analysis of CALB-CNTs-C=N-[bmim][N(Tf)$_2$] MR 1:0.1

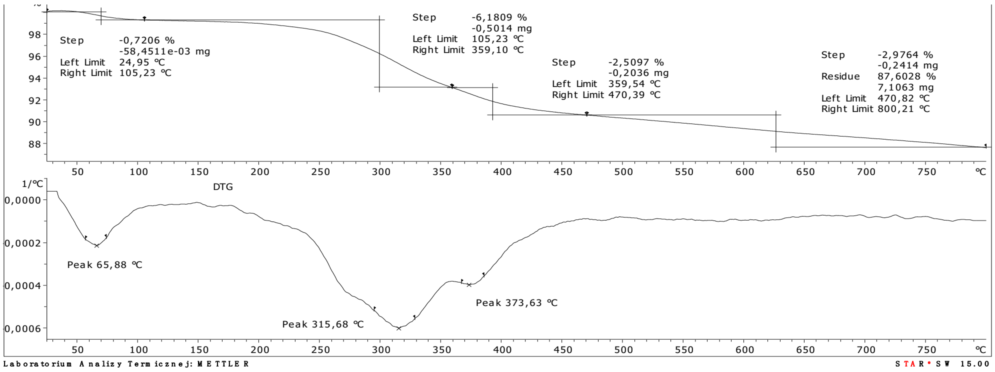

Figure S38. Thermogravimetric analysis of CALB-CNTs-C=N-[bmim][N(Tf)$_2$] MR 1:0.3

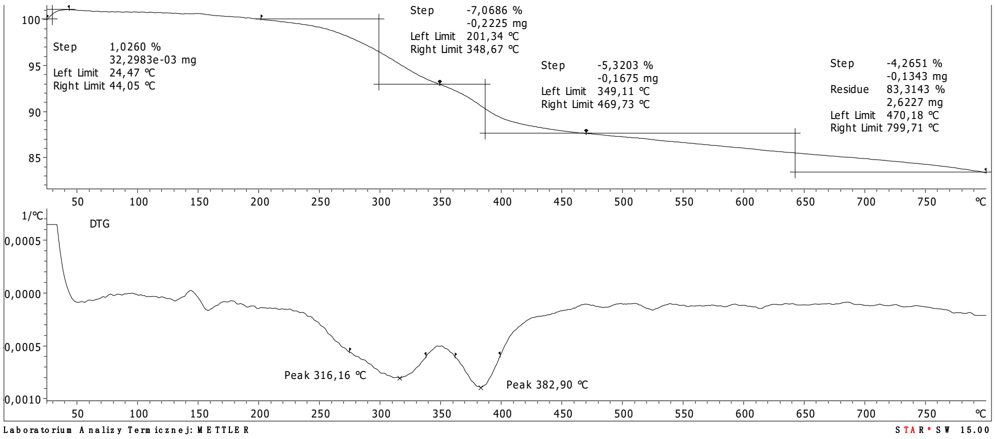

Figure S39. Thermogravimetric analysis of CALB-CNTs-C=N-[bmim][N(Tf)$_2$] MR 1:0.5

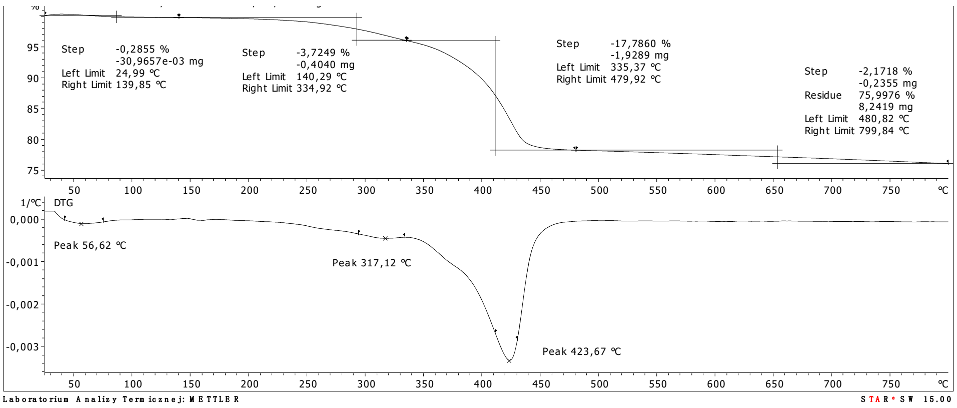

Figure S40. Thermogravimetric analysis of CALB-CNTs-C=N-[bmim][N(Tf)$_2$] MR 1:0.8

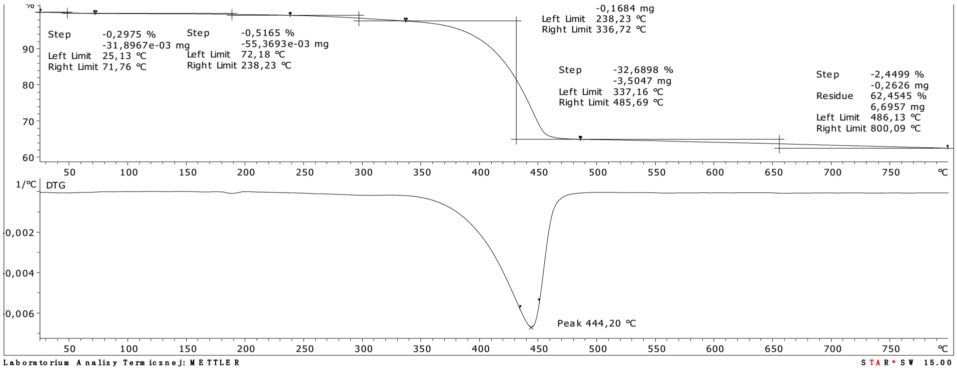

Figure S41. Thermogravimetric analysis of CALB-CNTs-C=N-[bmim][N(Tf)$_2$] MR 1:1.1

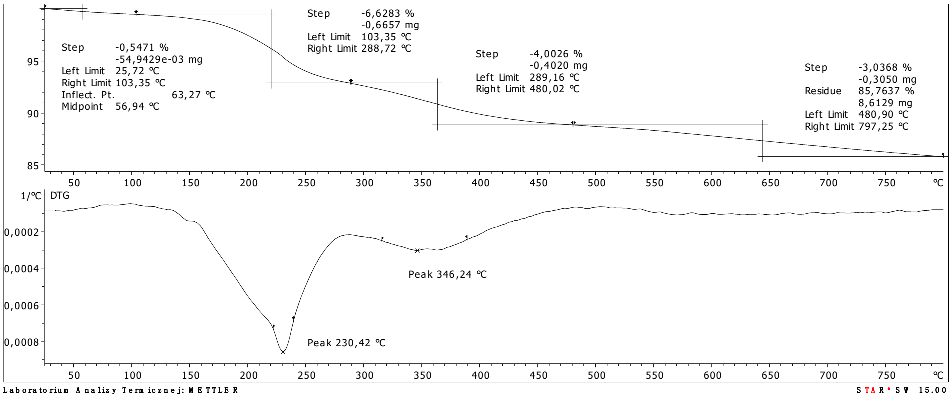

Figure S42. Thermogravimetric analysis of CALB-CNTs-CONH-Oc_2PO_4 after 4th reaction run.

Section S5. IR analysis

The crucial step of both pathways lies in a synthesis of a bond between MWCNTs and an amine. In case of incomplete substitution of functional groups, an activity of final biocatalyst could be lower than it would be possible to achieve. In order to determine types of bonds existing between atoms during a progress of the synthesis, the IR and XPS analyses were performed.

The results of IR analysis of structures A1 and I1 in comparison with starting materials were shown in Table S1 and Figures S43-S44. Performed experiments confirmed the final structures.

Table S1. Characteristic bands observed in FT-IR spectra.

FT-IR (KBr, cm^{-1})	
Aromatic structure of CNTs	3000 – 2800 stretching vibrations of C_{Ar}-H ($\nu_{C\text{-}H}$)<br>1660 – 1400 C=C groups in aromatic rings ($\nu_{C=C}$)
CNTs-CONH (**A1**)	3430 ($\nu_{N\text{-}H}$), 1630 (I amide $\nu_{C=O}$), 1512 (II amide $\delta_{N\text{-}H}$)<br>1232 ($\nu_{C\text{-}N}$), 664 ($\gamma_{N\text{-}H}$)
CNTs-CHO	2920 and 2850 ($\nu_{O=C\text{-}H,\ asym.}$), 1710 ($\nu_{C=O}$)
CNTs-C=N (**I1**)	1630 ($\nu_{C=N}$)

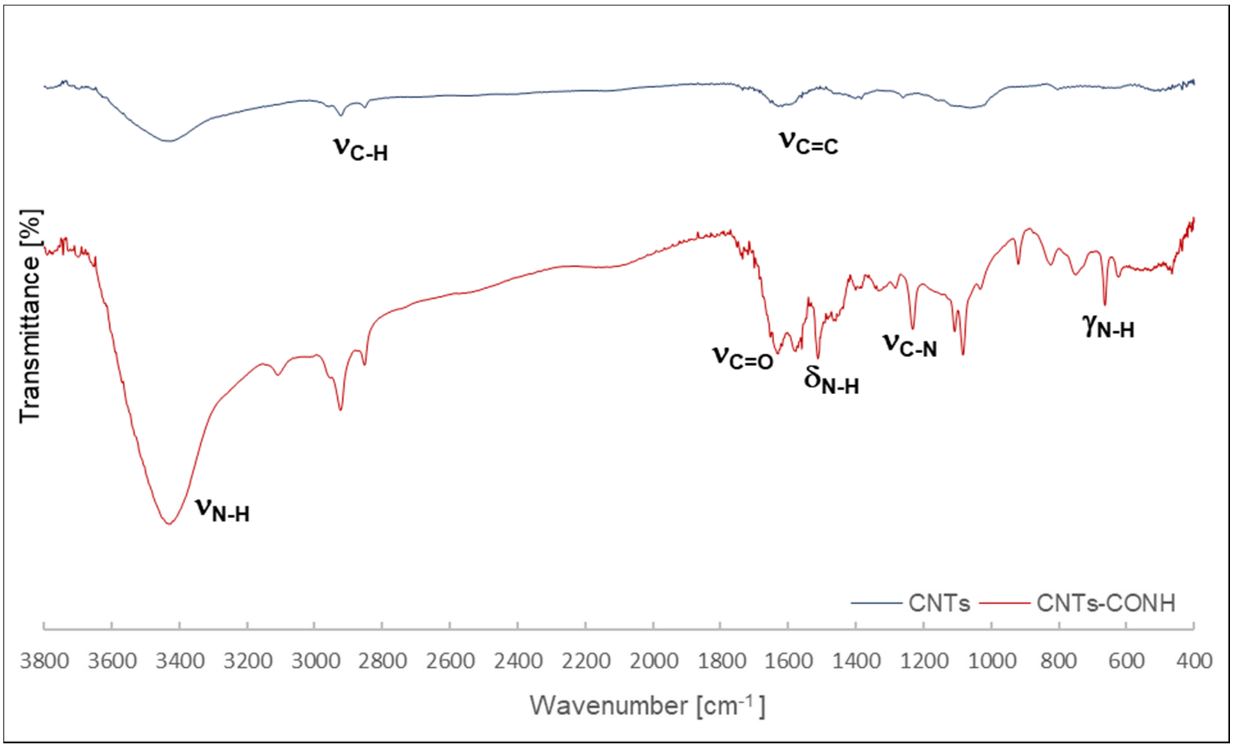

Figure 43. FT-IR spectra of pristine CNTs (blue) and CNTs-CONH (**A1**) (red).

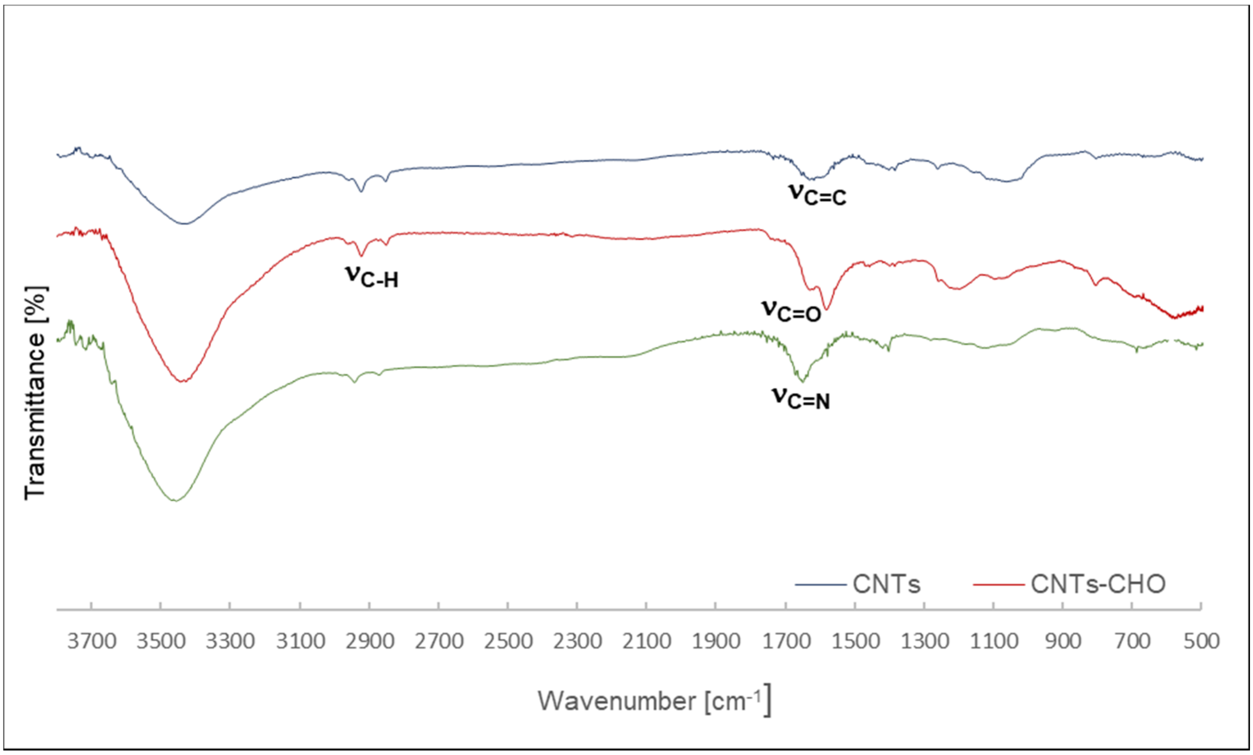

Figure 44. FT-IR spectra of pristine CNTs (blue), CNTs-CHO (red) and CNTs-C=N (I1) (green).

Section S6. XPS analysis

The results of XPS analysis of structures A1, A2, I1 and I2 were shown in Tables S2-S5 and Figures S45-S48. Performed experiments confirmed the final structures.

Table S2. XPS analysis of CNTs-CONH (A1).

Analysis	BE / eV	Atomic conc., %	interpretation of the structure
**C1s**	282.4	2.2	C-C
	284.5	46.1	C=C
	286.3	27.1	C-N
	287.8	5.3	C=O
**O1s**	530.7	0.6	O=C
	532.1	3.7	O-C
	534.1	0.6	O=C
	535.7	0.1	H-O-H from water adsorbed on the surface
**N1s**	397.4	0.6	N_2
	399.5	5.7	C-N from imidazolium ring
	401.3	5.5	C-NH from amide bond
	403.1	0.4	C=N from imidazolium ring

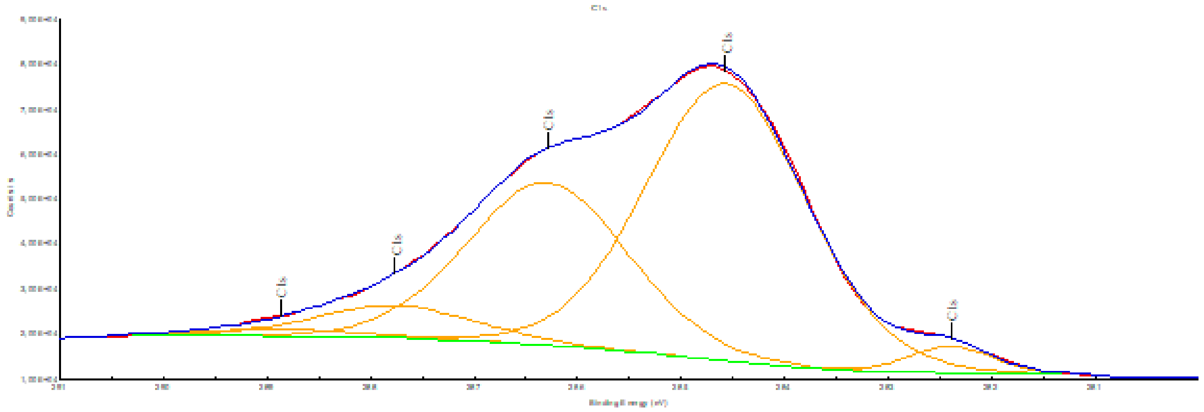

Figure S45. XPS analysis of CNTs-CONH (A1).

Table S3. XPS analysis of CNTs-CONH-HEX-I (A2).

Analysis	BE / eV	Atomic conc., %	interpretation of the structure
**C1s**	282.4	4.8	C-C
	284.5	71.3	C=C
	286.3	14.3	C-N
	287.8	2.5	C=O
**O1s**	531.1	1.4	O=C
	532.5	0.9	O-C
	533.8	0.6	O=C
	536.1	0.2	H-O-H from water adsorbed on the surface
**N1s**	397.8	0.1	N_2

	399.7	0.8	C-N from imidazolium ring
	401.7	1.6	C-NH from amide bond
	403.8	0.2	$C-N^+$ from imidazolium ring
I3d5	618.4	0.9	iodide (I^-)
	620.5	0.2	I_2

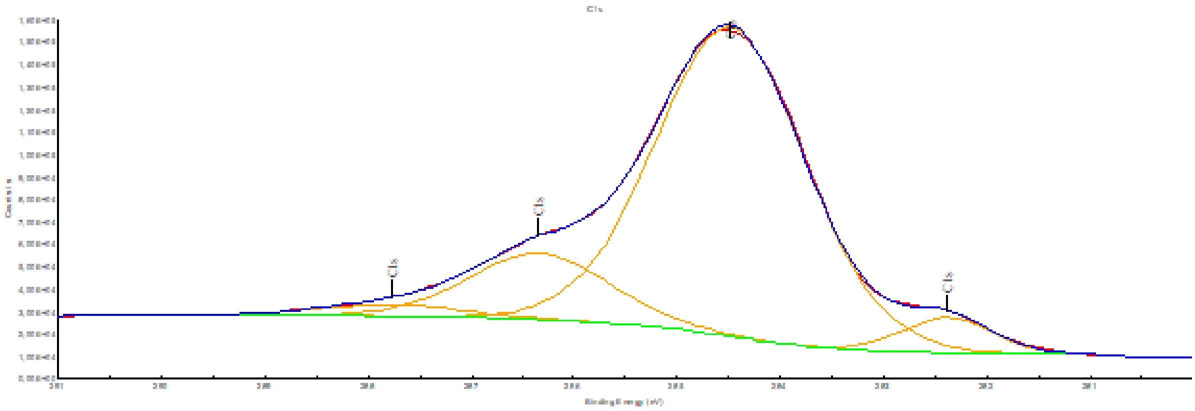

Figure S46. XPS analysis of CNTs-CONH-HEX-I (A2).

Table S4. XPS analysis of CNTs-C=N (I2).

Analysis	BE / eV	Atomic conc., %	interpretation of the structure
C1s	282.4	5.3	C-C
	284.5	74.8	C=C
	286.3	12.7	C-N
	287.4	2.6	C=N
O1s	530.8	0.9	O=C
	532.3	0.7	O-C
	533.7	0.5	O=C
	536.2	0.2	H-O-H from water adsorbed on the surface
N1s	399.2	1.0	C-N from imidazolium ring
	401.2	1.2	C=N from imine bond
	405.7	0.1	C=N from imidazolium ring

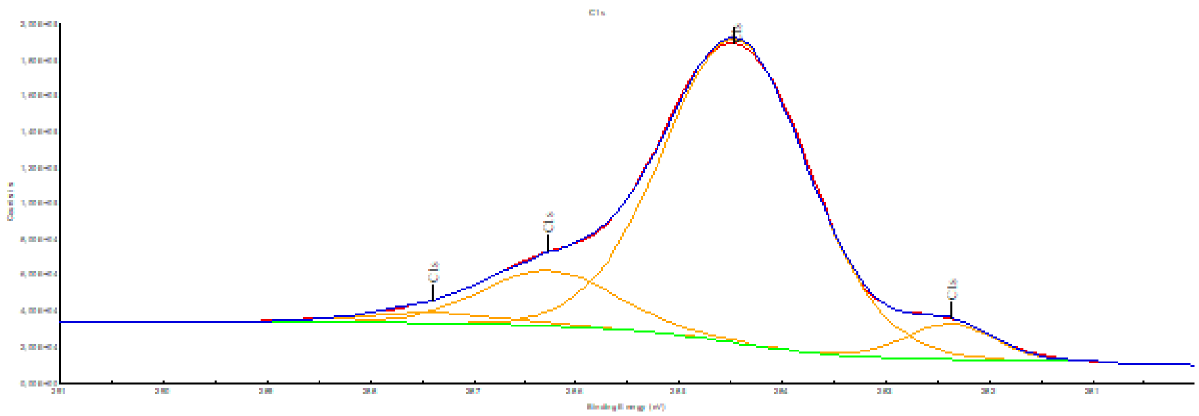

Figure S47. XPS analysis of CNTs-C=N (I2).

Table S5. XPS analysis of CNTs-C=N-HEX-I (I3).

Analysis	BE / eV	Atomic conc., %	interpretation of the structure
**C1s**	282.5	4.3	C-C
	284.5	77.0	C=C
	286.3	12.9	C-N
	287.7	1.2	C=N
**O1s**	531.1	1.2	O=C
	532.8	1.2	O-C
	534.3	0.5	O=C
	536.2	0.2	H-O-H from water adsorbed on the surface
**N1s**	399.1	0.2	C-N from imidazolium ring
	401.4	0.7	C=N from imine bond
	406.3	0.2	$C\text{-}N^{+}$
**I3d5**	618.4	0.3	iodide (I^{-})
	620.4	0.1	I_2

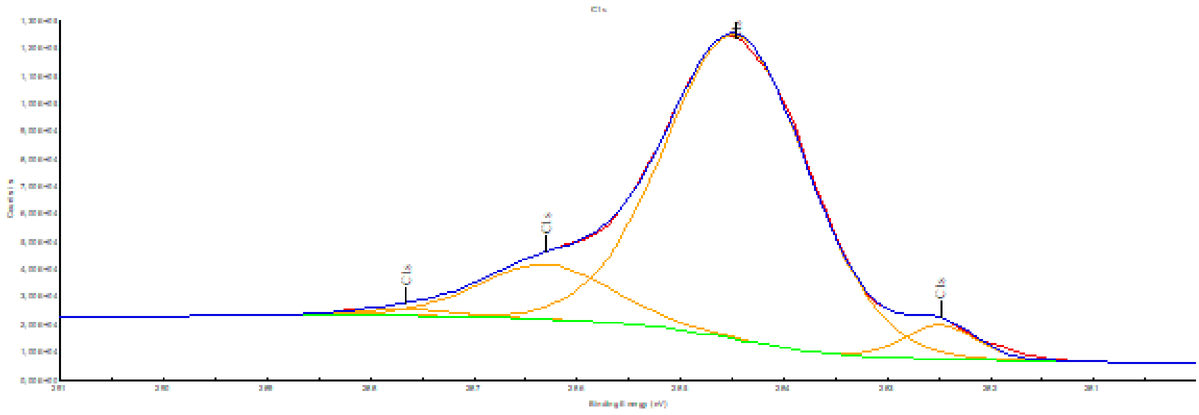

Figure S48. XPS analysis of CNTs-C=N-HEX-I (I3).

Section S7. Elemental analysis.

Table S6. Elemental analysis of synthesized structures.

Structure	Content of atoms: C, H, N (wt.%)
CNTs-COOH	98.12, 0.29, 0.23
CNTs-CONH (A1)	96.19, 0.65, 1.83
CNTs-CONH-HEX-I (A2)	91.83, 0.92, 1.21
CNTs-CONH-HEX-$N(Tf)_2$ (A3)	86.55, 0.94, 1.44
CNTs-CONH-HEX-$N(CN)_2$ (A4)	90.84, 0.95, 2.57
CNTs-CONH-HEX-Oc_2PO_4 (A5)	91.83, 0.92, 1.53
CNTs-CONH-HEX-$OcSO_4$ (A6)	88.75, 1.44, 1.49
CNTs-CHO	96.19, 0.48, 0.29
CNTs-C=N (I1)	95.05, 0.64, 1.01
CNTs-C=N-HEX-I (I2)	95.44, 0.52, 0.41
CNTs-C=N-HEX-$N(Tf)_2$ (I3)	91.46, 0.60, 0.91
CNTs-C=N-HEX-$N(CN)_2$ (I4)	91.61, 0.66, 2.47
CNTs-C=N-HEX-Oc_2PO_4 (I5)	96.39, 0.54, 0.44
CNTs-C=N-HEX-$OcSO_4$ (I6)	94.82, 0.52, 0.45

Section S8. Influence of the type of an enzyme on the recyclability of biocatalysts

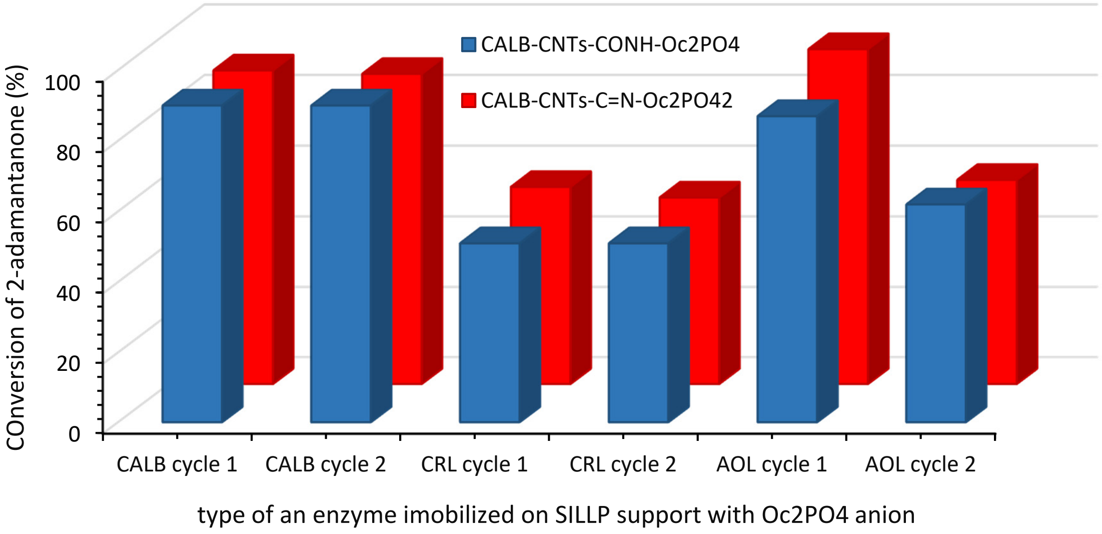

Figure S49. Influence of the type of an enzyme on the recyclability of biocatalysts. *Reaction conditions:* 2-adamantanone 1 mmol (0.150 g), toluene 0.5 mL, *n*-octanoic acid 0.5 mL, 0.080 g CALB-CNTs-CONH-Oc_2PO_4 or CALB-CNTs-C=N-Oc_2PO_4 biocatalyst, 35 % aq. H_2O_2 2 mmol (0.194 g), 20 °C, 4 h, 400 rpm.
